# Golgi-localized STELLO proteins regulate the assembly and trafficking of cellulose synthase complexes in *Arabidopsis*

**DOI:** 10.1038/ncomms11656

**Published:** 2016-06-09

**Authors:** Yi Zhang, Nino Nikolovski, Mathias Sorieul, Tamara Vellosillo, Heather E. McFarlane, Ray Dupree, Christopher Kesten, René Schneider, Carlos Driemeier, Rahul Lathe, Edwin Lampugnani, Xiaolan Yu, Alexander Ivakov, Monika S. Doblin, Jenny C. Mortimer, Steven P. Brown, Staffan Persson, Paul Dupree

**Affiliations:** 1 Max-Planck Institute for Molecular Plant Physiology, Am Muehlenberg 1, 14476 Potsdam, Germany; 2 Department of Biochemistry, University of Cambridge, Tennis Court Road, Cambridge CB2 1QW, UK; 3 Energy Biosciences Institute, and Plant and Microbial Biology Department, University of California, Berkeley, California 94720, USA; 4 School of Biosciences, University of Melbourne, Parkville, Victoria 3010, Australia; 5 Department of Physics, University of Warwick, Coventry CV4 7AL, UK; 6 Laboratório Nacional de Ciência e Tecnologia do Bioetanol (CTBE), Centro Nacional de Pesquisa em Energia e Materiais (CNPEM), Caixa Postal 6192, Campinas, São Paulo CEP 13083–970, Brazil; 7 ARC Centre of Excellence in Plant Cell Walls, School of Biosciences, University of Melbourne, Parkville, Victoria 3010, Australia

## Abstract

As the most abundant biopolymer on Earth, cellulose is a key structural component of the plant cell wall. Cellulose is produced at the plasma membrane by cellulose synthase (CesA) complexes (CSCs), which are assembled in the endomembrane system and trafficked to the plasma membrane. While several proteins that affect CesA activity have been identified, components that regulate CSC assembly and trafficking remain unknown. Here we show that STELLO1 and 2 are Golgi-localized proteins that can interact with CesAs and control cellulose quantity. In the absence of STELLO function, the spatial distribution within the Golgi, secretion and activity of the CSCs are impaired indicating a central role of the STELLO proteins in CSC assembly. Point mutations in the predicted catalytic domains of the STELLO proteins indicate that they are glycosyltransferases facing the Golgi lumen. Hence, we have uncovered proteins that regulate CSC assembly in the plant Golgi apparatus.

## Abstract (web summary)

Cellulose is produced in plants by cellulose synthase complexes (CSCs) that are assembled in the endomembrane system and then trafficked to the plasma membrane. Here Zhang *et al*. show that the Golgi-localized STELLO1 and 2 proteins are required for the proper assembly and distribution of CSCs in plant cells.

## Graphical abstract

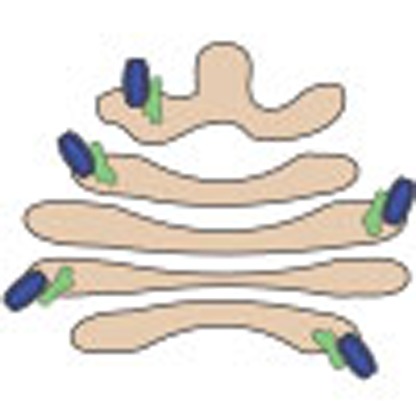

Plant cell walls are essential for plant growth and development, and protect cells against external stress^1^. During growth, plant cells are surrounded by a strong yet adaptable primary cell wall. Once growth has ceased, and depending on the function of the cell, an additional secondary wall may be deposited.

The bulk of plant cell wall polysaccharides are synthesized in the Golgi and secreted to the apoplast, with the exception of cellulose that is synthesized at the plasma membrane by cellulose synthase (CesA) complexes (CSCs)^2^,^3^. As the most abundant biopolymer on Earth, cellulose is a principal component of both primary and secondary cell walls. Genetic and biochemical studies have revealed that two hetero-trimers of CesA proteins (CesA1, 3, and the 6-like CesAs, as well as CesA4, 7 and 8) are involved in primary and secondary wall synthesis in *Arabidopsis thaliana*, respectively^4^,^5^,^6^. The CesA subunits are synthesized in the endoplasmic reticulum (ER), and CSC rosettes with sixfold symmetry have been observed in the Golgi, but it remains unclear where the CesA oligomerization and assembly into CSCs occurs^7^,^8^,^9^. The CSCs are trafficked to the plasma membrane via the trans-Golgi network (TGN), and/or via small post-Golgi vesicles that are known as small CesA compartments or microtubule-associated CesA compartments^10^,^11^. Once the CSCs are delivered to the plasma membrane they begin to synthesize cellulose microfibrils. During synthesis, the nascent microfibrils become embedded in the cell wall and the activity of the CSCs thus provides a motile force that drives the complexes forward through the membrane^12^. The direction of the CSC movement is guided by cortical microtubules, via associations with CesA interacting1 (refs 13, 14, 15).

Apart from the CesA proteins, several components that impact on cellulose synthesis have been identified, including the GPI-anchored protein COBRA, the endo-glucanase KORRIGAN, the chitinase-like proteins (CTL1 and 2) and the recently discovered companion of CesA1 and 2 (refs 16, 17, 18, 19, 20, 21). Most of these proteins affect the activity of the CSCs once they have reached the plasma membrane^3^. However, less is known about processes that influence cellulose synthesis in other cellular compartments. Proper maturation of N-linked glycans affects cellulose synthesis, as mutations in glucosidases (*knopf* and *rsw3*) and in a mannose-1-phosphate guanylyltransferase (*cyt1*) resulted in cell elongation defects and reduced cellulose levels^22^,^23^,^24^. More recent reports show that defects in the *N*-glycosylation machinery affect the glycosylation of the endo-glucanase KORRIGAN, and that mutations of the glycosylation sites led to reduced glucanase activity, and mis-localization, of the protein^25^,^26^,^27^. Nevertheless, N-linked glycosylation is a process that affects a substantial fraction of the cell's proteome, and components along the secretory pathway that distinctively affect the activity of CSCs therefore remain unknown.

Here we report on two Golgi-localized proteins, STELLO1 and 2 (STL1 and 2, classical Greek ‘to set in order, arrange, send'), that specifically affect cellulose production. STL1 and 2 can interact with the CesAs, and mutations in the proteins led to defects in CesA secretion and activity, and altered the distribution of the CesAs in the Golgi cisternae. We further demonstrate that the STLs affect CSC assembly, which impacts on cellulose synthesis efficiency.

## Results

### Mutations in STL1 and STL2 cause cellulose-related phenotypes

Genes that are co-expressed with the *CesA* genes tend to be associated with cellulose synthesis^28^. Using the pfam-based co-expression tool FamNet (http://aranet.mpimp-golm.mpg.de/famnet.html, ref. 29), we found that the pfam domain of unknown function (DUF)288 was co-expressed with the pfam CesA (Supplementary Fig. 1a). The DUF288 pfam contains two *Arabidopsis* proteins, At2g41770 and At3g57420, which we named STL1 and STL2. STL homologues are present throughout the plant kingdom, but STL proteins are distinct from distantly related proteins in nematodes, fungi and molluscs (Supplementary Fig. 1b). Microarray data suggested that *STL1* and *STL2* have similar expression profiles, and are active in cells that are expanding or producing secondary cell walls (Supplementary Fig. 1c), which we confirmed with transgenic *Arabidopsis* plants expressing *pSTL1:STL1-GFP* (Supplementary Fig. 1d–i).

**Figure 1:**
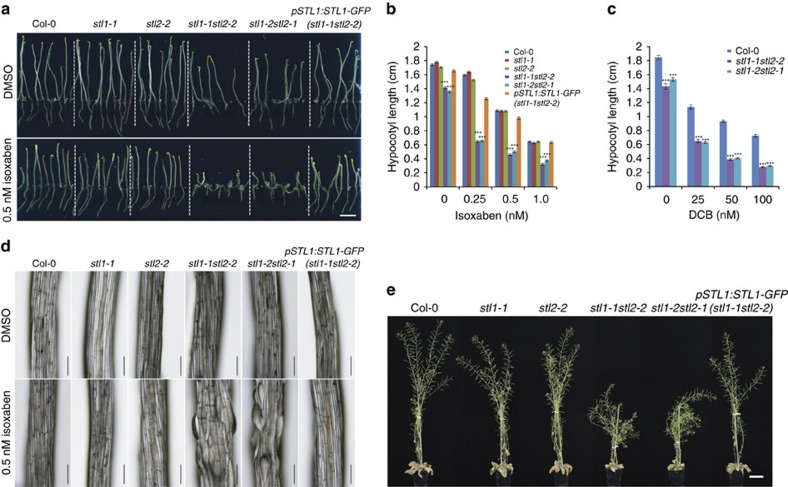
Mutations in STL1 and STL2 impact on plant growth. (**a**) Six-day-old Col-0, *stl1-1*, *stl2-2*, *stl1-1stl2-2*, *stl1-2stl2-1* and *pSTL1:STL1-GFP(stl1-1stl2-2*; STL1 fused C-terminally to GFP under its native promoter expressed in the *stl1-1stl2-2* mutant background) seedlings grown in the dark on half MS media (upper panel) or on half MS media supplemented with 0.5 nM isoxaben (lower panel). Scale bar, 0.5 cm. (**b**,**c**) Bar graphs of hypocotyl length on media supplemented with increasing concentration of isoxaben (**b**) or DCB (**c**). Values are mean (±s.e.) from three biological replicates with more than 10 seedlings per replicate. ***P value<0.001, Student's *t*-test. (**d**) Close-up of hypocotyl cells of seedlings grown as in **a** using stereo microscopy. Scale bar, 200 μm. (**e**) Eight-week-old greenhouse grown plants. Scale bar, 5 cm.

Homozygous *stl* T-DNA insertion lines (*stl1-1*, SALK_029852; *stl1-2*, GABI_733B10; *stl2-1*, SALK_023535; and *stl2-2*, SALK_095790) that expressed no full-length *STL1* and *STL2*, respectively (Supplementary Fig. 2a–c), did not differ in growth compared with wild-type plants (Fig. 1a–e). To test whether the STLs are functionally redundant, we generated *stl1stl2* double mutants (*stl1-1stl2-2* and *stl1-2stl2-1)*. Both the primary roots of light-grown seedlings and etiolated hypocotyls of the *stl1stl2* mutants were significantly shorter compared with wild-type (Fig. 1a–c; Supplementary Fig. 2d–f). In addition, 8-week-old soil-grown *stl1stl2* mutant plants exhibited stunted growth (Fig. 1e).

**Figure 2:**
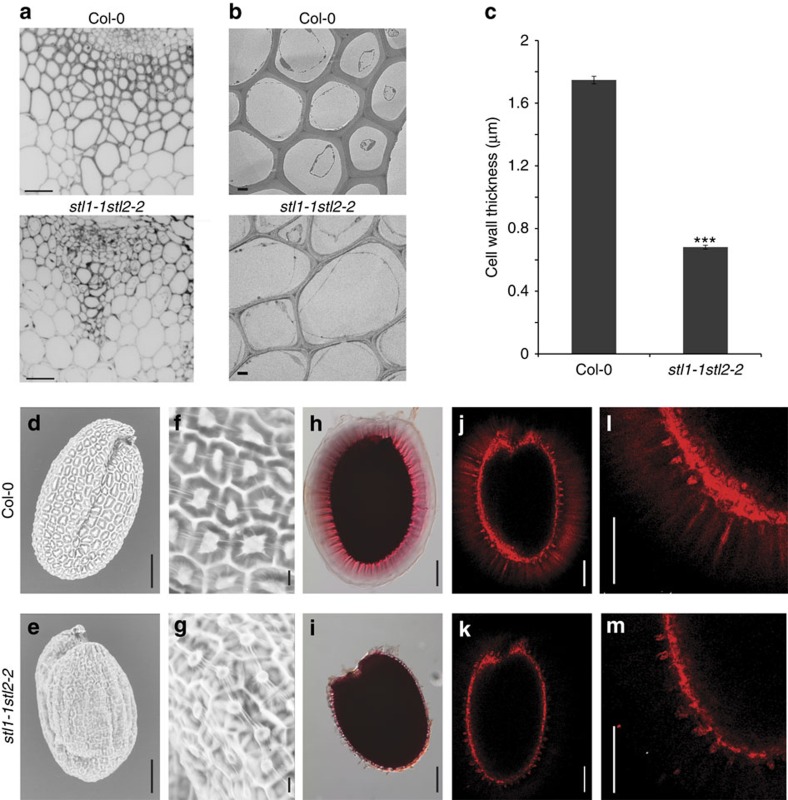
Mutations in STL1 and STL2 affect secondary cell walls and seed mucilage. (**a**) Confocal images of stem sections from the stem base of six-week-old greenhouse grown plants stained with calcofluor white. Scale bar, 50 μm. (**b**) TEM of basal stem fibre cell walls of sections equivalent of **a**. Scale bar, 2 μm. (**c**) Secondary wall thickness measured between two adjacent cells in TEM images as in **b**. Error bars represent s.e., *n*≥498 cells from four plants. ***P value<0.001, Student's *t*-test. (**d**–**g**) Scanning electron microscope images of seeds, showing aberrant columella shape in *stl1stl2* mutants. (**h**–**m**) Adherent mucilage stained with ruthenium red (**h**,**i**) and cellulose stained with pontamine fast scarlet 4B (**j**–**m**). Scale bars, 100 μm in **d**,**e**,**h**–**m**; scale bars, 10 μm in **f**,**g**.

Primary wall cellulose-related mutants display increased sensitivity to cellulose synthesis inhibitors^3^,^5^,^21^. The *stl1stl2* mutants were similarly hypersensitive, displaying severe cell swelling, in response to either isoxaben or 2,6-dichlobenzonitrile (DCB; Fig. 1a–d; Supplementary Fig. 2d–f). The STL proteins also affected secondary wall production, as the *stl1stl2* mutants showed occasional collapsed xylem vessels, and the interfascicular fibre cell-wall thickness was substantially reduced (Fig. 2a–c), which can also be observed in secondary wall cellulose synthesis mutants^30^. Cellulose synthesis is also important in seed columella development, and cellulose contributes to rays in the seed mucilage adherent layer^31^,^32^,^33^,^34^. Indeed, seed columella shape was abnormal, and the cellulosic rays and adherent mucilage were absent in the *stl1stl2* mutants (Fig. 2d–m). Our data thus indicate that *stl1stl2* mutant plants show widespread impairment in cellulose production.

### The *stl1stl2* mutant contains less cellulose

**Figure 3:**
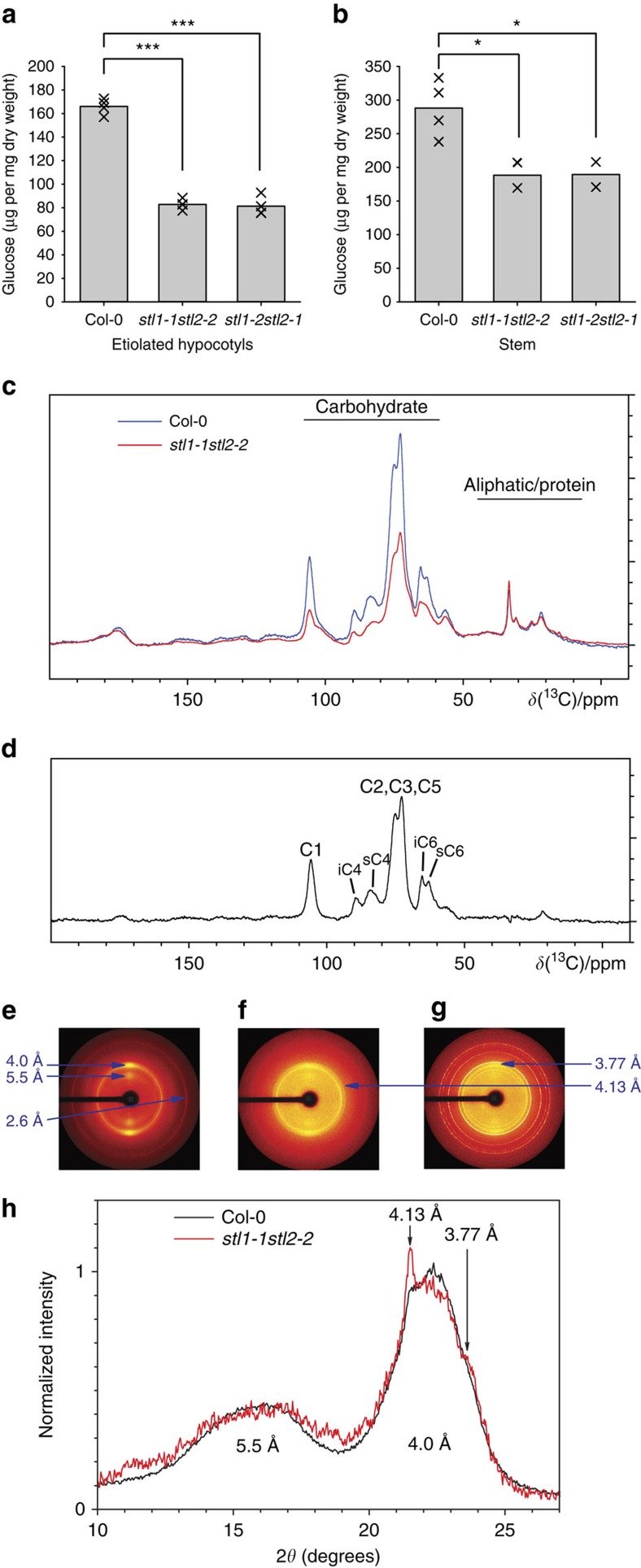
Cellulose levels are reduced in the *stl1stl2* mutant. (**a**,**b**) Cellulose content of primary cell walls (**a**; six-day-old etiolated seedlings, ∼300–400) and secondary cell walls (**b**; six-week-old greenhouse-grown stem material, >10 stems). Individual values and the mean from four (**a**) and two (**b**) biological replicates are shown. *P value<0.05, ***P value<0.001, Student's *t*-test. (**c**) ^1^H (850 MHz)-^13^C solid-state cross-polarization-MAS NMR spectra of wild-type and *stl1stl2* stems. The signal intensity was normalized to the aliphatic/protein region (35–25 p.p.m.) with the assumption that proteins and waxes were not substantially changed in the mutant. (**d**) Subtracted ^13^C solid-state NMR spectrum (wild-type-*stl1stl2*) to reveal signals cellulose carbons C1–C6 specifically reduced in *stl1stl2*. The microfibril surface (s) and internal (i) C4 and C6 are labelled. (**e**–**g**) X-Ray fibre diffraction patterns from wild-type (**e**) and *stl1stl2* mutants with lower (**f**) or higher (**g**) spurious diffraction intensities attributed mainly to wax. Arrows in **e** point to line corresponding to d-spacing of native cellulose (5.5, 4.0 and 2.6 Å). Arrows in **f**,**g** point to rings corresponding to d-spacing of wax (4.13 and 3.77 Å). The 4.13-Å ring is also visible in **e**. (**h**) Normalized diffraction intensity along the equatorial direction after subtraction of CRAFS-resolved background. The diffractograms are averages from three (wild-type) and two (*stl1stl2*) biological replicates. Only diffractions patterns with lower spurious signal (such as the one in **f**) were included in the average *stl1stl2* diffractograms presented in **h**.

To confirm that cellulose levels were reduced in the *stl1stl2* mutants, we measured cellulose content by glucose release using Saeman hydrolysis. Indeed, the cellulose content was reduced by ∼50% in the young hypocotyls of *stl1stl2* mutants, indicating that primary wall cellulose synthesis was significantly reduced (Fig. 3a). Additionally, cellulose was reduced by ∼40% in secondary cell-wall-rich stems of *stl1stl2* mutants (Fig. 3b). The solid-state ^13^C cross-polarization-magic angle spinning (MAS) nuclear magnetic resonance (NMR) spectra of intact *Arabidopsis* stems showed substantial changes in the carbohydrate region of the mutant (Fig. 3c,d). Subtraction of the mutant spectrum from the wild-type spectrum showed loss of cellulose in the *stl1stl2* mutant, consistent with the assessment by Saeman hydrolysis. Nevertheless, the peaks of cellulose carbons, including crystalline cellulose carbon 4 at 89 p.p.m. were still visible in the mutant. The X-ray diffraction pattern of the *stl1stl2* mutant showed less intensity than the wild type (Fig. 3e–h; Supplementary Fig. 3a–c), possibly due to the thinner mutant stems and reduced cellulose content. After background subtraction and intensity normalization, the mutant radial diffraction profile was only subtly different to that of the wild type, with a broader peak arising from the 5.5-Å d-spacing (Fig. 3h; Supplementary Fig. 3a,b), which comprises the (1–10) and (110) reflections indexed according to the Iβ crystal structure^35^. Since the strategy for background subtraction was designed to reduce the differences between the wild type and the mutant radial diffraction profiles, the actual differences may be greater than they appear in these profiles. Such fine differences in cellulose diffraction profiles are inherently difficult to interpret. We speculate that heterogeneity of cellulose environments and the altered proportions between cellulose and matrix polysaccharides might be contributing to the observed changes in diffraction profiles. Hence, these different approaches all indicate that the *stl1stl2* mutant contained lower levels of cellulose as compared with wild type. In contrast, the composition and relative proportion of the matrix-phase polysaccharides did not appear to be substantially altered as determined by the amount of monosaccharides released by trifluoroacetic acid (TFA) hydrolysis of alcohol-insoluble residues of both etiolated hypocotyls and stems (Supplementary Table 1).

### STL1 and STL2 are localized to the Golgi

**Figure 4:**
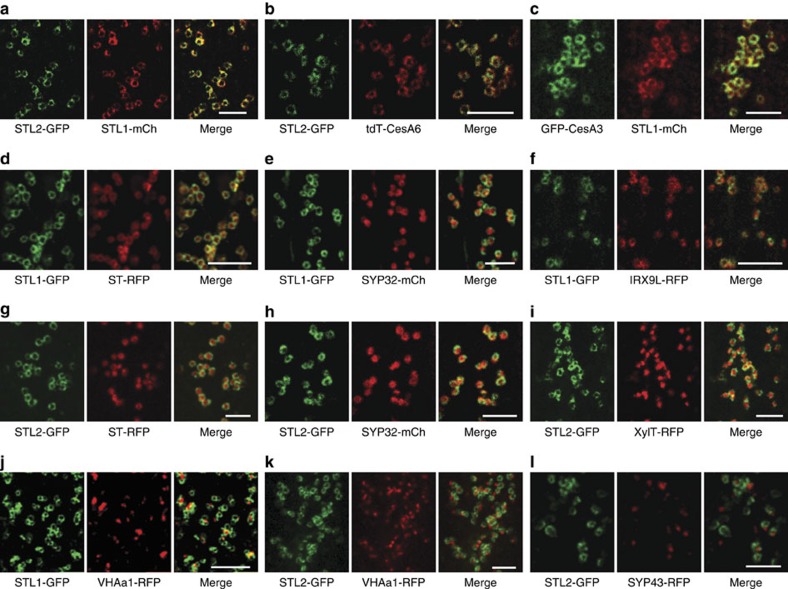
STL1 and STL2 are localized to the Golgi. Individual images of a 3-day-old etiolated hypocotyl cell expressing both STL1-mCherry and STL2-GFP (**a**), STL2-GFP and tdTomato-CesA6 (**b**), GFP-CesA3 and STL1-mCherry (**c**), STL1-GFP and ST-RFP (**d**), STL1-GFP and SYP32-mCherry (**e**), STL1-GFP and IRX9L-RFP (**f**), STL2-GFP and ST-RFP (**g**), STL2-GFP and SYP32-mCherry (**h**), STL2-GFP and xylosyltransferase-RFP (**i**), STL1-GFP and VHAa1-RFP (**j**), STL2-GFP and VHAa1-RFP (**k**), STL2-GFP and SYP43-RFP (**l**). Scale bar, 5 μm.

To evaluate where in the cell the STLs may function, we generated *pSTL1:STL1-GFP, pUb10:STL1-mCherry* and *pUb10:STL2-GFP* constructs. These constructs were functional as they partially restored growth of the *stl1stl2* mutant (Fig. 1a–e; Supplementary Fig. 4a,b). We observed green fluorescent protein (GFP) and mCherry signals in ring-shaped cytosolic compartments that resembled Golgi in etiolated hypocotyls (Fig. 4a). Dual-labelled STL2-GFP and STL1-mCherry plants revealed that the two STLs co-localized in the ring-shaped compartments (Fig. 4a; Supplementary Fig. 4c).

To see if the ring-like compartments represented Golgi, we introgressed different red fluorescent protein (RFP)- or mCherry-tagged Golgi and TGN markers into the STL1- and STL2-GFP lines, respectively. We also crossed the STL-FP lines with plants expressing tdTomato-CesA6 (tdT-CesA6) or GFP-CesA3 to investigate possible co-localization of the proteins. The dual-labelled seedlings showed that STL-GFP proteins did not co-localize well with the TGN markers vacuolar ATPase subunit a1 (VHAa1)-RFP and SYP43-RFP (Fig. 4j–l; Supplementary Fig. 4c), but did show partial co-localization with the trans-Golgi marker sialyltransferase (ST)-RFP and the cis-Golgi marker SYP32-mCherry, and with the Golgi proteins beta-1,2-xylosyltransferase (XylT)-RFP and IRX9L-RFP (Fig. 4d–i; Supplementary Fig. 4c). Furthermore, the STL proteins co-localized well with tdT-CesA6 and GFP-CesA3 (Fig. 4b,c; Supplementary Fig. 4c), supporting a cellulose-related function of the STLs in the Golgi apparatus.

### A STL glycosyltransferase-like domain is in the Golgi lumen

**Figure 5:**
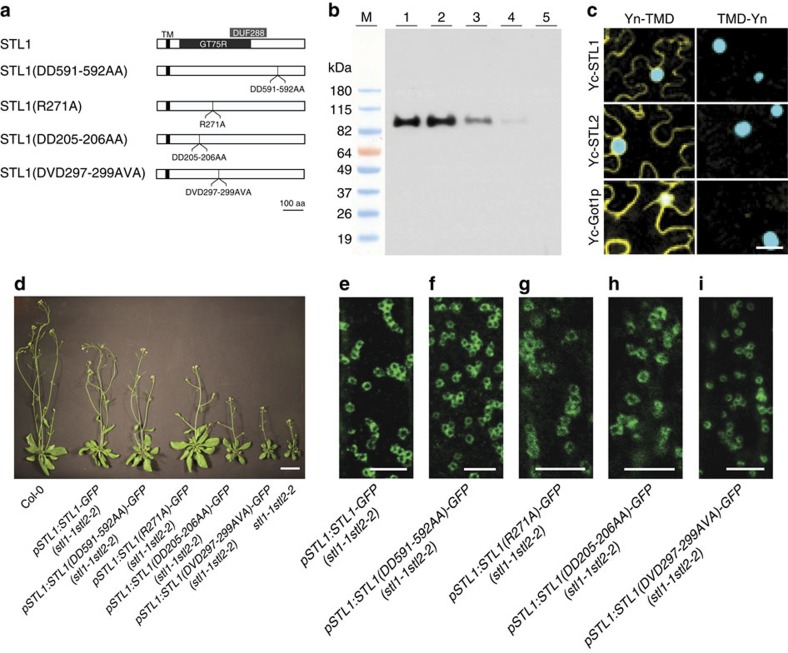
The STL proteins hold a glycosyltransferase-related domain that faces the Golgi lumen and that is important for STL function. (**a**) Schematic diagram to show the putative transmembrane domain (TM), the regions with homology to GT75 (GT75R) and DUF288 of the STL proteins, and the point mutations used in **d**–**i**. (**b**) Protease protection assay using microsomes prepared from *Arabidopsis* callus expressing the STL1-myc protein. STL1-myc protein was detected by western blot using an anti-myc antibody. Lanes: 1. no detergent/no protease, 2. no detergent/proteinase K, 3. Triton 0.1%/proteinase K, 4. Triton 0.5%/proteinase K, 5. Triton 1%/proteinase K. M, molecular weight markers. (**c**) *N. benthamiana* epidermal leaf cells expressing Yn-TMD (cytosolic reporter) or TMD-Yn (Golgi lumen reporter) together with Yc-STL1, Yc-STL2 and Yc-Got1p, respectively. Yc, C-terminal part of VENUS; Yn, N-terminal part of VENUS; TMD, the first 52 amino acids of the rat ST protein. Yellow signal indicates that the two proteins interact and therefore are facing the same side of the Golgi membrane. The nuclear marker CFP-N7 (cyan) was included as a positive transformation control in all experiments. Scale bar, 50 μm. (**d**) Six-week-old *stlstl2* mutants expressing STL1-GFP or point mutants DD591-592AA, R271A, DD205-206AA and DVD297-299AVA of the STL1-GFP gene fusion. Note that only DD591-592AA and R271A complemented the *stl1stl2* mutant. Scale bar, 2 cm. (**e**–**i**) Individual images of 3-day-old etiolated hypocotyl cells expressing either of the STL1-GFP (**e**), DD591-592AA (**f**), R271A (**g**), DD205-206AA (**h**) and DVD297-299AVA (**i**). Scale bar, 5 μm.

The STL proteins contain a region of homology to proteins of the CAZy glycosyltransferase (GT) 75 family that includes the self-glycosylating protein UDP-arabinose mutase (RGP), suggesting they might possess glycosylation activity (Fig. 5a; Supplementary Fig. 5a, discussed in ref. 36). Hydrophobicity analysis suggested that the STLs contain a single transmembrane domain near their N-termini and have a type II topology with their C-termini in the Golgi lumen, like most Golgi GTs (Fig. 5a). To assess how the STLs are oriented in the Golgi membrane, we performed microsomal protease protection assays in the absence or presence of detergent to disrupt the membranes. In intact microsomes, the fusion protein was resistant to protease, but was digested if detergent was added, indicating that the C-terminus of the protein resides within the Golgi lumen (Fig. 5b). To confirm these results, we performed a bimolecular-fluorescence complementation (BiFC)-based Golgi protein membrane topology (GO-PROMTO) assay^37^. The N-terminal part of VENUS (Yn; the first 155 amino acids), or the C-terminal part of VENUS (Yc; the last 84 amino acids), was fused in frame either before (cytosolic reporter) or after (Golgi luminal reporter) the first 52 amino acids of the rat ST protein (TMD), which consists of a transmembrane domain targeted to the Golgi membrane (Supplementary Fig. 5b). Clear fluorescence complementation was observed on co-expression of both Yn/Yc-STL1 and Yn/Yc-STL2 with the cytosolic reporter, but not with the luminal reporter (Fig. 5c; Supplementary Fig. 5c). These results corroborate that the N-terminus of STL proteins faces the cytoplasm.

To investigate what parts of the protein are relevant for the functions, we generated truncated protein versions where either the C-terminal or N-terminal parts of the STL1 were removed (Supplementary Fig. 5d). Both the C-terminal and N-terminal constructs were still localized to compartments reminiscent of Golgi, but only the C-terminal end of the protein was able to restore growth of the *stl1stl2* mutant seedlings on isoxaben-containing media (Supplementary Fig. 5e–i). The C-terminal construct was also localized to the peripheral part of Golgi, while the N-terminal construct did not (Supplementary Fig. 5e,f). To investigate if the putative GT domain was important for STL function, we generated point mutations of amino acids that might contribute to the GT activity (Fig. 5a; Supplementary Fig. 5a). Mutations of conserved aspartates in the DXD motif putative sugar nucleotide binding region (DD205-206AA and DVD297-299AVA) impaired the function of the STLs, as the constructs were unable to complement *stl1stl2* mutant growth (Fig. 5d). However, the conserved arginine corresponding to a self-glycosylation site in RGPs (R271A), and aspartates elsewhere in the protein (DD591-592AA) were not required as the mutated proteins restored *stl1stl2* plant growth. The inability of some of the STL-GFP constructs to complement was not due to changes in protein localization, as all of the proteins were visible as ring-like Golgi compartments (Fig. 5e–i). Therefore, STL membrane topology and site-directed mutagenesis experiments are consistent with STL proteins having a GT activity in the Golgi lumen.

### STL1 and STL2 can interact with the CesA proteins

**Figure 6:**
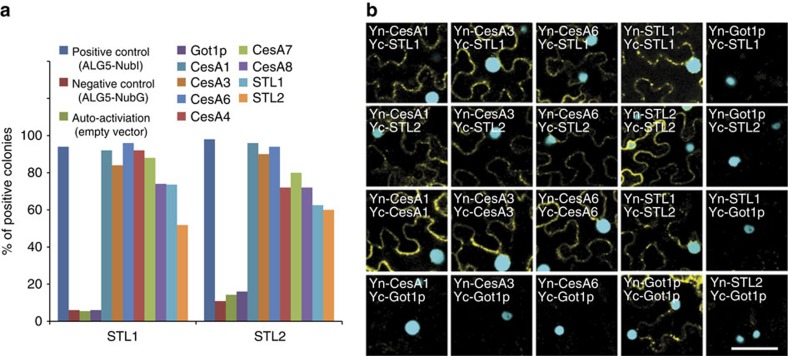
STL1 and STL2 can interact with the CesA proteins. (**a**) Split-ubiquitin assays using STLs as bait and CesAs as prey in yeast. Values are the percentage of yeast colonies that displayed growth after 4 days on selective medium at 28 °C. ALG5 (yeast ALG5 dolicholphosphoglucose synthetase) was fused with wild-type N-terminal part of ubiquitin (NubI) as positive control. Mutated N-terminal part of ubiquitin (NubG) was used in the rest of the vectors including the auto-activation control (empty vector), negative control (ALG5 and Got1p) and CesAs. (**b**) BiFC assays detecting the interaction of CesAs and STLs in *N. benthamiana* epidermal leaf cells. The N-terminal (Yn) or C-terminal (Yc) part of VENUS was fused in frame with CesA1, CesA3, CesA6, STL1, STL2 and Got1p (negative control), respectively. The combination of constructs is indicated in each figure panel. The nuclear marker CFP-N7 (cyan) was included as a positive transformation control in all experiments. Scale bar, 50 μm.

The *stl1stl2* cellulose defects and the co-localization of the tdT-CesA6 with STL2-GFP and GFP-CesA3 with STL1-mCherry prompted us to investigate whether the proteins could interact. First, we used STL1 and STL2 as bait and the primary and secondary wall CesAs as prey proteins (that is, CesA1, 3, 4, 6, 7 and 8) in the split-ubiquitin-based membrane yeast two-hybrid system. Yeast growth on selective media supported an interaction between the proteins (Fig. 6a). In addition, we observed that the STL1 and STL2 could interact with each other to potentially form hetero- or homodimeric protein complexes (Fig. 6a).

We confirmed the yeast-based interactions by BiFC assays in tobacco epidermal leaf cells. As for the yeast assay, we used the Got1p *Arabidopsis* homologue (also identified as Wave line 18; At3g03180) as a negative control. Got1p localizes to the Golgi apparatus and has a similar membrane topology as that of the STLs (Fig. 5c; ref. 38). The N- (Yn) or C- (Yc) terminal part of VENUS was fused to the N-terminus of STL1, STL2, CesA1, CesA3, CesA6 and Got1p. We observed interactions between the CesAs and STL1 or STL2 (Fig. 6b; Supplementary Fig. 6a,b), but not between the Got1p and the CesAs or the STL proteins. In addition, the STL and Got1p proteins showed homo-dimerization capability, which served as a positive control for the constructs (Fig. 6b; Supplementary Fig. 6b). Our data thus support that the CSC can interact with the STL proteins.

### STLs influence the Golgi localization of CesA3 protein

**Figure 7:**
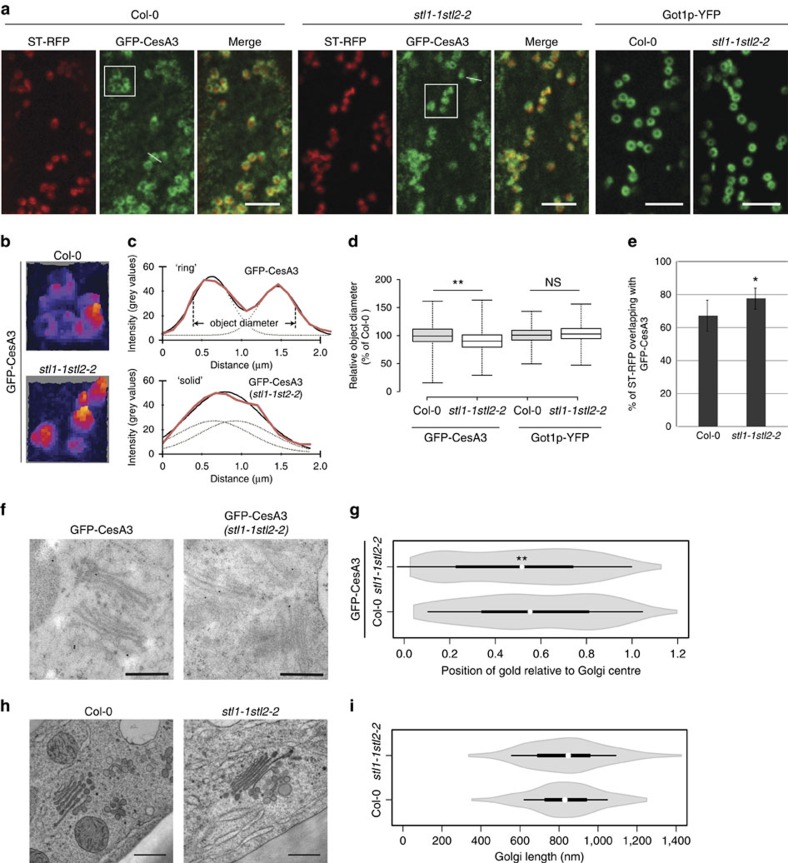
Mutation of STLs changes the CesA distribution in Golgi. (**a**) Images of 3-day-old etiolated hypocotyl cells expressing ST-RFP and GFP-CesA3, or Got1p-YFP in Col-0 or *stl1stl2* mutant. Scale bar, 5 μm. (**b**) 3D heat-map renderings of fluorescence intensity of the GFP-CesA3 signal (white rectangles in **a**). (**c**) Relative fluorescent signal intensity across transects in **a**. Black solid lines indicate fitting of a double-Gaussian distribution to the intensity sections (red lines). Object diameter was calculated by summing the peak-to-peak distance and the width of the individual Gaussians (dotted lines). (**d**) Relative object diameter of GFP-CesA3 or Got1p-YFP in Col-0 and *stl1stl2* mutant. Centre lines equal medians; box limits equal 25th and 75th percentiles; whiskers display minimum and maximum values. For GFP-CesA3, *n*=870 Golgi (25 cells from 12 control plants); *n*=820 Golgi (26 cells from 13 *stl1-1stl2-2* plants). For Got1p-YFP, *n*=567 Golgi (16 cells from 8 control plants); *n*=583 Golgi (14 cells from 7 *stl1-1stl2-2* plants). **P value<0.01, Student's *t*-test; NS, not significant. (**e**) Quantitative estimates of co-localization (Mander's coefficient) of GFP-CesA3 and ST-RFP in Col-0 or *stl1stl2* seedlings. *n*≥7 cells per genotype. *P value<0.05, Student's *t*-test. (**f**) Representative TEM images of immuno-gold staining of GFP-CesA3 in wild-type and *stl1stl2* plants. Scale bar, 500 nm. (**g**) Violin plots of Golgi gold periphery distributions. Values show the distance of each gold particle from the centre, relative to the total length, of the Golgi. *n*≥190 gold particles per genotype from two independent immunoTEM experiments. **P value<0.01, Student's *t*-test. (**h**) Representative TEM images for Golgi morphology in Col-0 and *stl1stl*2 plants. Scale bar, 500 nm. (**i**) Violin plots of Golgi lengths. *n*=200 Golgi per genotype from three independent cryofixation experiments. No significant difference between the wild-type and *stl1stl2* plants was observed (*P*=0.76, Student's *t*-test). White circles show medians in **g**,**i**; box limits equal 25th and 75th percentiles; whiskers extend 1.5 times the interquartile range from the 25th and 75th percentiles; polygons represent density estimates of data and extend to extreme values.

The observed interaction of the STL proteins and the CesAs suggested that the STLs could influence the CSCs in the Golgi. Interestingly, the Golgi distribution of the GFP-CesA3 fluorescence was changed in the *stl1stl2* mutant as compared with wild type (Fig. 7a–e). The typical ring-shaped GFP-CesA3 signal, prominent in wild-type cells, was less pronounced in the *stl1stl2* mutant cells and instead resembled solid fluorescent spheres (Fig. 7a–c). We observed much more of the Golgi-localized GFP-CesA3 signal as solid spheres in the *stl1stl2* (54% of 820 Golgi) as compared with wild type (8% of 870 Golgi scoring scheme as outlined in Fig. 7c). In addition, quantification of the fluorescence distribution revealed a significant reduction of object diameter in *stl1stl2* mutants compared with the control (Fig. 7d). Surprisingly, similar changes in fluorescence distribution were not observed for the Golgi-localized protein Got1p-YFP nor for IRX9L-RFP which are localized to the Golgi periphery (Fig. 7a,d; Supplementary Fig. 6c,d). Moreover, co-localization analyses of the ST-RFP and GFP-CesA3 in the *stl1stl2* mutant revealed improved coincidence of the two fluorophores (Fig. 7e), corroborating a specific change of the GFP-CesA3 Golgi distribution in the *stl1stl2* mutant.

We confirmed the altered CesA Golgi distribution by performing immuno-gold labelling with anti-GFP and a gold-conjugated secondary antibody in GFP-CesA3-expressing plants. Gold labelling, representing GFP-CesA3, was detected at the plasma membrane demonstrating specific labelling (Supplementary Fig. 6e). Gold label was also found in the Golgi apparatus (Fig. 7f). Similar to the live-cell imaging results, we found that GFP-CesA3 was confined to a more narrow Golgi localization in *stl1stl2* as compared with that of wild type (Fig. 7f,g; Supplementary Fig. 6f,g). Importantly, no significant differences in Golgi shape, nor in the number of Golgi cisternae, were observed in the *stl1stl2* mutant as compared with wild type (Fig. 7h,i; Supplementary Fig. 6h,i).

### STLs impact CSC assembly and plasma membrane motility

**Figure 8:**
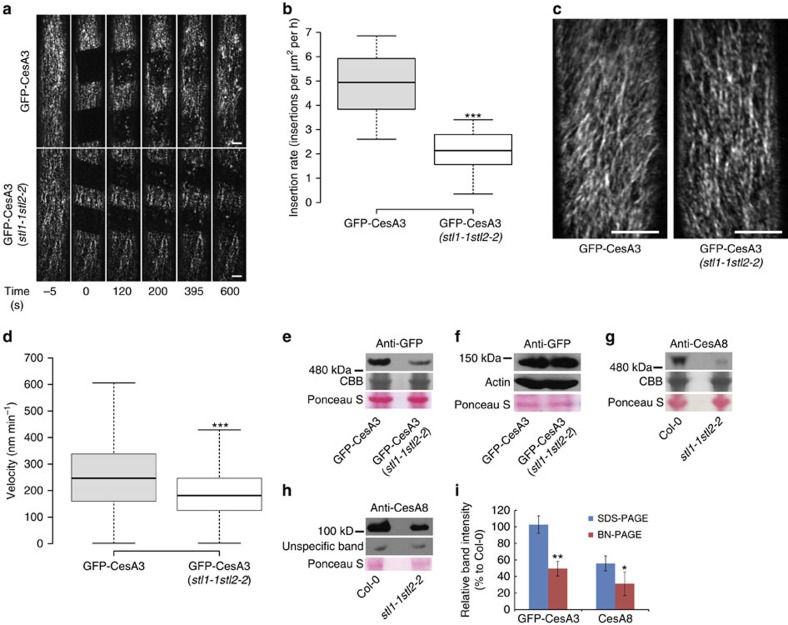
STL function is necessary for CSC dynamics and assembly. (**a**) Photo-bleaching of three-day-old wild-type or *stl1stl2* hypocotyl cells expressing GFP-CesA3. Scale bar, 5 μm. (**b**) Box plot displaying re-population of the plasma membrane with fluorescent CesA foci (given as mean delivery rates of CesAs per area unit per hour). *n*≥11 cells from more than 6 seedlings per genotype. ***P value<0.001, Student's *t*-test. (**c**) Time average images of GFP-CesA3 at the plasma membrane focal plane of 3-day-old etiolated Col-0 and *stl1stl2* mutant cells. Scale bar, 10 μm. (**d**) Box plot of GFP-CesA3 speed estimates from cells such as those in **c**. *n*≥10 cells from 4 seedlings per genotype. ***P value<0.001, Student's *t*-test. In both **b** and **d**, centre lines show the medians; box limits indicate the 25th and 75th percentiles; whiskers extend to minimum and maximum values. (**e**–**h**) Protein amount of CesA and CSC in wild-type and *stl1stl2* mutants. Fourteen-day-old wild-type and *stl1stl2* mutants expressing GFP-CesA3 (**e**,**f**) or stems of 5-week-old wild-type and *stl1stl2* mutants (**g**,**h**) were used for microsome preparation (**e**,**g**) or total protein extraction (**f**,**h**). The samples were analysed by BN-PAGE (**e**,**g**) or SDS-PAGE (**f**,**h**) followed by western blot with anti-GFP (**e**,**f**) or anti-CesA8 antibody (**g**,**h**). Ponceau S staining of the membranes, coomassie blue (CBB) staining of protein gels, western blot with anti-actin or unspecific bands are shown for protein loading control. (**i**) Relative band intensity of western blot in *stl1-1stl2-2* samples (normalized with the band intensity in control). Values are mean (±s.e., *n*=3). **P value<0.01 and *P value<0.05, Student's *t*-test.

To investigate whether the CesA behaviour was further affected in the *stl1stl2* mutant, we first assessed the secretion of the CesAs by fluorescence recovery after photo-bleaching (FRAP) experiments of GFP-CesA3 at the plasma membrane focal plane^14^. Only punctate structures in the central subregion were counted to avoid false positives due to active CesA complexes migrating into the region of quantification. Our FRAP experiments revealed that the CesAs are typically delivered with a rate of 4.8±1.2 fluorescent CesA foci per μm^2^ per hour (±indicates s.d., *n*≥11 cells from more than 6 seedlings per genotype) in wild-type hypocotyl cells (Fig. 8a,b). However, in the *stl1stl2* double mutant this rate was substantially reduced (2.2±0.9 fluorescent CesA foci per μm^2^ per hour; Fig. 8a,b).

Plasma membrane-based motility of fluorescently labelled CesAs is typically used as a proxy for complex activity^13^. To assess complex activity we therefore also measured the GFP-CesA3 movement at the plasma membrane using Imaris analyses of spinning-disc confocal image series^15^. We found that while the GFP-CesA3 proteins typically migrate with a speed of 255±128 nm min^−1^ (±indicates s.d., *n*≥10 cells from 4 seedlings per genotype) in wild-type hypocotyls, the speed was significantly reduced in the *stl1stl2* mutants to 191±92 nm min^−1^ (Fig. 8c,d).

The changes of CSC behaviour in the *stl1stl2* mutant suggested that the STL proteins might affect the CSC assembly in the Golgi. To investigate this we performed blue-native polyacrylamide gel electrophoresis (BN-PAGE) analyses of microsomes from seedlings, or plant stems, of wild type and the *stl1stl2* mutants. The CSCs can be seen as multimeric protein complexes when analysed by BN-PAGE (refs 9, 39). Indeed, we detected bands at high molecular weight (Fig. 8e,g); however, the intensity of these bands was substantially reduced in the *stl1stl2* mutants as compared with wild-type plants (Fig. 8e,g; Supplementary Fig. 7a). Similar reductions in band intensities were observed for both primary (anti-GFP; GFP-CesA3), and secondary (CesA8 antibodies), wall CSCs in the *stl1stl2* mutants (Fig. 8e,g,i; Supplementary Fig. 7a). In contrast, the GFP-CesA3 levels were not, and the CesA8 levels only moderately, reduced in the *stl1stl2* mutant in the SDS-PAGE analysis (Fig. 8f,h,i; Supplementary Fig. 7a). This reduction was significantly less prominent as compared with the multimeric CSC band intensities (Fig. 8e–i). Hence, relatively less CesAs are incorporated into multimeric CSCs in the *stl1stl2* mutant as compared with wild type. Our data therefore indicate that the STL proteins regulate the CSC assembly, which influences the secretion and activity of the CSCs.

## Discussion

Cellulose is of immense importance to plant growth and as raw material for industries. However, very little is known about when and how the CSCs are assembled and trafficked to the plasma membrane. Here, we report on the Golgi-localized STL proteins that can interact with the CesAs, and that impact on Golgi distribution, secretion and activity of the CSC by affecting its assembly.

Our data indicate that the STLs are GTs that act in the lumen of Golgi apparatus. Loss of STL function changed the spatial distribution of the CSCs in the Golgi. However, we did not observe similar changes of other Golgi-located proteins, such as Got1p and IRX9L. These data support a certain specificity of the STLs towards the CSCs, which was corroborated by our observations that the STLs and the CesA proteins can interact. While reports on altered spatial distributions of proteins within Golgi stacks remain scarce, a recent study proposes that cargo proteins are excluded from the central region of the trans-Golgi cisternae^40^. The confinement of the CSCs to the central region of the Golgi in the *stl1stl2* cells would thus imply that they are not properly set for secretion, supporting that the GT-related activity of the STLs is necessary for accurate CSC assembly. Indeed, the CesAs were less well incorporated into multimeric CSCs, and the delivery rate of CSCs to the plasma membrane was reduced in the mutant. In addition, the CSC activity at the plasma membrane was impaired and X-ray fibre diffraction observations revealed that the cellulose crystals synthesized might have altered structure in the mutant as compared with wild type, corroborating that the CSCs are not properly arranged in the *stl1stl2*.

**Figure 9:**
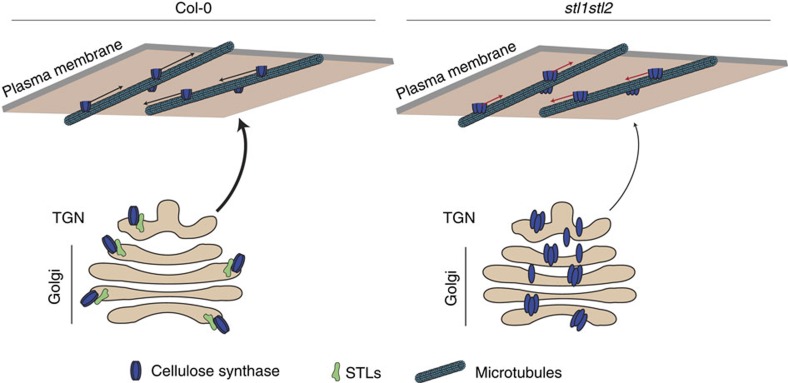
Schematic model of how the STL proteins function in cellulose production. In growing wild-type cells, the STL proteins are located to the Golgi. Here the proteins control the assembly of the CSCs. Once the CSCs are delivered to the plasma membrane they begin to synthesize cellulose. In the absence of the STL proteins the assembly of the CSCs is impaired, which cause aberrant localization of the complexes in the Golgi and that lead to defects in CSC trafficking to, and activity at, the plasma membrane.

The STLs could also have a role in maintaining the CSCs in an inactive state until they have reached the plasma membrane. If so, the STLs and the CesAs would be delivered to the cell surface together. Here, the CSCs ought to become activated and the STLs would therefore need to be effectively removed from the plasma membrane. Indeed, we did not observe any detectable fluorescence associated with STL-GFP at the plasma membrane, supporting such a function. However, if the CSCs were active at the Golgi, as then would be the case in the *stl1stl2* mutants, we would expect severe defects in Golgi structure and function due to aberrant cellulose deposition, which we did not observe in our transmission electron microscopy (TEM) studies. We therefore favour a scenario in which the STL proteins affect the assembly of the CSCs (Fig. 9).

It is plausible that the STLs could glycosylate the CSC or associated components that impact on CSC function and localization. Glycosylation of CesA proteins has not been reported, and they are not thought to be N-glycosylated^22^, but non-conventional types of protein glycosylation of CesAs cannot be ruled out. In contrast, KORRIGAN, which is closely associated with the CSC, has N-glycosylations important for function^26^,^27^. While it is perhaps unlikely that a GT75-related GT would be involved in modifying these types of glycans, it is possible that the STLs could affect glycans attached to KORRIGAN. This could be mediated via a direct interaction with KORRIGAN, or via interactions with other GTs in the Golgi, which in turn could lead to defects in cellulose synthesis as observed in the *stl1stl2* mutants. However, we did not see any differences in protein size or the cellular location of GFP-labelled KORRIGAN in the *stl1stl2* mutant as compared with wild type (Supplementary Fig. 7b,c). While these data do not rule out the possibility that the STLs function on KORRIGAN, it appears more likely that they act on other components of the CSC machinery.

Our results support a model in which the STL proteins regulate the assembly of the CSCs, and perhaps maintain the CSCs in a certain state during their transit from the Golgi to the plasma membrane (Fig. 9). Perturbations in the actin cytoskeleton, V-ATPase activity at the TGN and lesions in the kinesin FRA1 do affect the trafficking of the CSC to the plasma membrane^41^,^42^,^43^. However, these defects affect a range of other cellular processes and specific components that regulate trafficking and integrity of the CSCs have therefore been wanting. Hence, the STLs open new avenues into understanding the function and regulation of cellulose synthesis and provide a cornerstone in how we comprehend a plant's ability to regulate this process.

## Methods

### Plant materials and growth condition

T-DNA insertional lines for *stl1-1* (SALK_029852), *stl1-2* (GABI_733B10), *stl2-1* (SALK_023535) and *stl2-2* (SALK_095790) were obtained from NASC (http://*Arabidopsis*.info/; ref. 44). Primers used for genotyping are listed in Supplementary Table 2. The pCesA6:tdTomato-CesA6, pCesA3:GFP-CesA3, VHAa1-RFP, ST-RFP, xylosyltransferase-RFP, SYP43-RFP, GFP-KOR and Got1p-YFP marker lines were described previously^5^,^27^,^40^,^45^,^46^,^47^,^48^,^49^. The pCesA3:GFP-CesA3 line in *je5* mutant background was also crossed with *stl1-1stl2-2* and the segregating plants were used for analysing the behaviour of GFP-CesA3 in control and *stl1stl2* mutants.

*Arabidopsis* plants were grown on half mass spectrometry (MS) media with 1% sucrose in long day condition (16 h light and 8 h dark) or dark. For drug treatments, the seeds were directly germinated and grown for indicated days on plates supplemented with various amounts of Isoxaben (Dr Ehrenstorfer GmbH) or DCB (Sigma).

### Sequence analysis

The amino acid sequence of *Arabidopsis* STL1 was used to search the publicly available databases at NCBI (http://www.ncbi.nlm.nih.gov/) and Phytozome (http://phytozome.jgi.doe.gov/pz/portal.html). The identified protein sequences from *A. thaliana, Populus trichocarpa, Brachypodium distachyon, Selaginella moellendorffii, Physcomitrella patens, Lottia gigantea, Mixia osmundae, Minthostachys verticillata, Ostreococcus tauri, Caenorhabditis elegans and Chlamydomonas reinhardtii* were trimmed and aligned with the MUSCLE algorithm. An unrooted phylogenetic tree was built with MEGA 6.06 (http://www.megasoftware.net/) using the maximum-likelihood Le and Gascuel (LG) model with Gamma distributed Invariant sites. One-thousand bootstrap replicates were completed to evaluate branch support length. The multiple sequence alignment based on predicted secondary structures of STL and RGP1 was performed by using the HHpred algorithm (http://toolkit.tuebingen.mpg.de/hhpred).

### Constructs

The ORFs of *STL1* and *STL2* were amplified from a seedling complementary DNA (cDNA) library with primer pairs STL1_for/STL1_rev and STL2_for/STL2_rev, respectively, and cloned into pENTR using the pENTR/D-TOPO Cloning Kit (Invitrogen, USA). For pUb10:STL2-GFP construct, London resin (LR) reactions of the Gateway cloning system (Invitrogen) were performed to sub-clone error-free STL2 into pUBC-GFP-DEST (ref. 50). The GFP fragment in pUBC-GFP-DEST was replaced by mCherry with restriction enzymes SpeI and PsiI, which was then used for LR reactions with STL1-pENTR to generate pUb10:STL1-mCherry construct.

The 1,000 bp STL1 upstream sequence including the 5′-UTR and the coding region including introns were amplified via primers pSTL1_for/pSTL1_rev and cloned into a pGreenII0000 vector containing the Oleosin-GFP from pFAST-G01 as selection marker^51^. The sequence was inserted between the *Apa*I at the 5′ and *Sal*I at the 3′. The stop codon was removed to C-terminally fuse in-frame the mGFP and a nopaline synthase (NOS) terminator. The mGFP was amplified with primers mGFP_for/mGFP_rev and cloned between *Sal*I at the 5′ and *Not*I at the 3′, the NOS terminator was amplified with primers NOS_for/NOS_rev and inserted between *Not*I at the 5′ and *Sac*I at the 3′. The point mutations were generated by Genescript.

For the split-ubiquitin assays, the CDS of STL1 and STL2 were amplified with primer pairs (STL1_pTFB1_f/STL1_pTFB_r and STL2_pTFB1_f/STL2_bait_rev, respectively) and cloned into the bait vector pTFB1 (Dualsystems Biotech AG, Switzerland) via the restriction enzyme sites *Stu*I and *Spe*I. The constructs with CesA1, 3, 6, 4, 7 and 8 in the prey vector pADSL-Nx were described previously^52^,^53^.

BiFC constructs were generated by amplifying PCR products using primers specified in Supplementary Table 2 and cloning them into *Sfo* I and *BamHI* linearized vectors pAMON (a modified pGREEN II binary vector containing the first 155 amino acids of VENUS driven by CaMV35S promotor) and pSUR (C-terminal version containing the last 84 amino acids of VENUS) using the Gibson assembly method^54^. To improve the signal and the signal-to-noise ratio of the above systems, amino acid 152 in VENUS was changed from isoleucine to leucine, as this lowers self-assembly when co-expressed with VC155. All generated constructs were sequence-verified and transformed into *Agrobacterium tumefaciens* strain AGL1 by electroporation with the helper plasmid pSOUP.

### Cross-section and staining of stems

Sections of basal mature stem were fixed and embedded in LR White as described below for electron microscopy. Sections were stained with 0.1% methylene blue and viewed on an Olympus BX61 microscope.

### Cell wall composition analysis

The basal part of the inflorescence stems from 6-week-old plants (5 cm from the base of the stem) were incubated in 96% (v/v) ethanol at 70 °C for 30 min and homogenized by ball milling (RETSCH Mixer Mill MM 400). The alcohol-insoluble residue was successively washed with absolute ethanol, twice with 2:3 (v/v) chloroform:methanol, and once with 65% (v/v), 80% (v/v) and absolute ethanol each and air-dried at room temperature. The monosaccharide composition analysis of the matrix cell wall polysaccharides was performed by high performance anion exchange (HPAEC) of acid hydrolysed cell walls. The alcohol-insoluble residue from stems (100 μg) was incubated in TFA (2 M, 1 ml) for 1 h at 121 °C. AIR of hypocotyls (1 mg) was hydrolysed by heating in 1.45 ml 4% sulfuric acid (121 °C, 60 min). Glucose in cellulose was measured after hydrolysis in 72% sulphuric acid at room temperature for 30 min and 1 M sulphuric acid for 3 h followed by neutralization with excess barium carbonate. The monosaccharide samples were separated using a Carbopac PA20 column on a HPAEC-PAD system (Dionex BioLC)^55^. The experiment was repeated three times, on three sets of independently grown plants.

### Electron microscopy

Sections of basal mature stem were fixed in 4% (w/v) formaldehyde, 1% (v/v) glutaraldehyde, 80 mM Pipes-HCl (pH 6.9) at 4 °C overnight. Following dehydration in ethanol, the sections were embedded in London Resin (LR) white and allowed to polymerize at 60 °C. Thin sections were prepared on EM grids using a microtome, and samples were viewed using a FEI Philips CM 100 TEM (Multi-Imaging Centre, University of Cambridge, UK).

Three-day-old etiolated hypocotyls were high-pressure frozen, freeze-substituted, embedded and sectioned according to McFarlane *et al*.^56^. Briefly, samples were cryofixed using a Leica HPM 100 in B-type sample holders (Ted Pella) with 1-hexadecene (Sigma) as a cryoprotectant. For morphology, samples were freeze substituted in 2% osmium tetroxide (Electron Microscopy Sciences) and 8% 2, 2-dimethoxypropane (Sigma) in acetone. Freeze substitution in a Leica AFS2 held samples for 5 days at −85 °C, after which samples slowly warmed to room temperature over 2 days, and were infiltrated with Spurr's resin over 4 days. Samples were sectioned to ∼70 nm using a Leica UCT microtome, suspended on copper grids (Gilder) coated with 0.3% formvar (Electron Microscopy Sciences), stained with 2% uranyl acetate in 70% methanol and Reynolds' lead citrate, then viewed on a Zeiss EM910 at 80 kV and images were collected using an Olympus Quemesa CCD camera. Golgi properties were quantified using ImageJ and the blind image analysis plugin BAR. Violin pots were generated using the BoxPlotR tool^57^.

For immunoTEM cryofixation, freeze substitution and embedding were performed as above, except that freeze substitution was performed with 0.25% glutaraldehyde, 0.1% uranyl acetate and 8% 2, 2-dimethoxypropane (Sigma) in acetone and samples were embedded in LR White resin (London Resin Company). Sections (70 nm) were suspended on nickel grids (Gilder) with 0.3% formvar and immunolabelling was performed according to McFarlane *et al*.^56^; samples were blocked with 5% bovine serum albumin in tris-buffered saline tween buffer (TBST; 10 mM Tris, 250 mM NaCl, 0.1% Tween20, pH 7.4) for 30 min, washed three times in TBST, then incubated in the primary antibody, 1/100 rabbit polyclonal anti-GFP (Life Technologies, A6455), diluted in TBST with 1% BSA for 1 h at room temperature. After 5 washes with TBST, samples were incubated in secondary antibody, 1/100 goat-anti-rabbit conjugated to 18 nm gold (ImmunoResearch Inc), diluted in TBST with 1% BSA for 1 h, then washed 3 times with TBST and 3 times with dH_2_O. Samples were post-stained with uranyl acetate and Reynolds lead citrate as above. Samples were viewed using a Philips CM120 BioTWIN at 120 kV and images were collected using a Gatan MultiScan 791 CCD camera. Gold particle distribution was measured using ImageJ and the blind image analysis plugin BAR.

For scanning electron microscopy of the seed columella layer, the dry mature seeds were mounted on surface of aluminium stubs and subsequently sputter coated with 10 nm of gold, and viewed using a Philips FEI XL30-FEG SEM operated at 5 kV (Multi-Imaging Centre, University of Cambridge, UK).

### MAS solid-state NMR

MAS solid-state NMR experiments were performed on a widebore Bruker (Karlsruhe, Germany) AVANCE III 850 MHz solid-state NMR spectrometer operating at 20T, corresponding to ^1^H and ^13^C Larmor frequencies of 850.2 and 213.8 MHz, respectively, using a 4-mm double-resonance MAS probe. Experiments were conducted at room temperature at a MAS frequency of 12.0 kHz±5 Hz. The ^13^C cross-polarization-MAS spectra were acquired using a 1-ms ramped contact time, a spectral window of 100 kHz (468 p.p.m.), a recycle delay of 2 s and 28,800 transients were co-added for each sample. The ^1^H 90° pulse duration was 3.4 μs. Two-pulse phase-modulated decoupling was applied during acquisition at a ^1^H nutation frequency of 83 kHz (ref. 58). The ^13^C chemical shift was determined using the carbonyl peak at 177.8 p.p.m. of L-alanine as an external reference with respect to TMS. Two independent stem samples were analysed.

### X-ray diffraction

*Arabidopsis* stems were aligned perpendicular to the CuKα X-ray beam (*λ*=1.5418 Å) generated by a Rigaku ultraX-18HF rotating anode with VariMax HR monochromating optics. Diffraction patterns were collected by a mar345 image plate positioned 200 mm behind the specimens. Instrumental line broadening and scattering angle 2*θ* were calibrated with an α-alumina reference sample. The CRAFS model was employed to resolve the isotropic polynomial background contributing to each diffraction pattern, thus separating the intensity arising from cellulose crystals^59^. In CRAFS modelling, we first obtained crystal parameters (unit cell, crystal size and peak profile shape) from analysis of Col-0 and then these crystal parameters were imposed for *stl1stl2* mutant. With this strategy, background subtraction attenuates differences between Col-0 and *stl1stl2*, thus assuring that remaining differences are not background subtraction artifacts.

Confirmatory experiments (Supplementary Fig. 3) with additional biological replicates were performed with the same modelling and X-ray detection set-up installed in the MX1 beamline (*λ*=1.4535 Å) of the Brazilian Synchrotron Light Laboratory^60^.

### Fluorescence imaging and analysis

The sub-cellular localization and dynamic behaviour of GFP, RFP or mCherry-tagged proteins were imaged with a confocal microscope equipped with a CSU-X1 Yokogawa spinning disc head fitted to a Nikon Ti-E inverted microscope, a CFI APO TIRF × 100 numerical aperture 1.47 oil immersion objective and an evolve charge-coupled device camera (Photometrics Technology). Image acquisitions were performed using Metamorph online premier (version 7.5). Seedling samples were mounted in half MS media between a cover glass and a 1-mm-thick agar (1%) pad affixed on a circular cover slip (Roth), thus stabilizing the sample and preventing it from compression and mechanical damage.

The M1 & M2 coefficients were analysed for co-localization quantification using the JACoP plugin^61^. Parameters were adjusted according to the specifications of the microscope and the optimal detection thresholds were manually determined by choosing the threshold at which the most objects and least noise pixels were found. The velocity of GFP-CesA3 at plasma membrane was calculated in Imaris software (Bitplane)^15^.

The 3D heat-map and line plot of fluorescence intensity was analysed in ImageJ (http://imagej.nih.gov/). To measure the object diameter of Golgi bodies, their cross-sectional intensity profiles were measured in ImageJ and analysed using a custom-made Matlab programme (Supplementary Data 1). The script used a peak-finding algorithm to determine the number of peaks in a cross-section. When the script found one or two peaks, we termed these Golgi bodies as ‘solid' and ‘ring', respectively. For solid Golgi body, we assumed that the two Gaussians were too closely positioned to be resolved individually. Thus, we fitted the cross-sections with a double-Gaussian distribution with the distance between the peaks being (i) a free parameter for ring-shaped Golgi and (ii) fixed to the resolution limit of our microscope (estimated to 266 nm) for solid Golgi. This approach yielded accurate estimations of the positions and widths of the two Gaussians. We defined the object diameter as the sum of the distance between the two peaks and the widths of each Gaussian.

### FRAP assays

FRAP experiments of GFP-CesA3 in control and *stl1stl2* mutants were performed using a FRAP system iLAS (Roper Scientific) integrated into the spinning-disk set-up^21^. The fluorescence at the PM was photobleached using the 488-nm laser and the delivery of new CesA particles was detected every 5 s for a period of 10 min. We scored the insertion events based on two criteria: first, before the delivery, the immediate region surrounding the delivery should be devoid of any CesA fluorescent particles, and second, after insertion, the particles should show the characteristic CesA tracking behaviour in subsequent frames as examined using kymographs. New CesA insertion events were identified in the central regions to avoid the CesAs moving from outside into the photobleached area.

### Split-ubiquitin-based Y2H

Interactions of STL1 and STL2 with different CesAs were analysed using the the split-ubiquitin MbYTH system^52^,^53^. Plasmids (200 ng each) for prey and bait were mixed and transformed into the yeast strain NYM51 (HYBRiGeNiCS). Positive transformants were selected with the SD media lacking Leucine and Tryptophan. The interactions of bait and prey were assessed by detecting the growth rate of at least 50 spotted yeast colonies after 4 days on s.d. media lacking Leucine, Tryptophan, Histidine and Adenine, and supplemented with 150 mM 3-ammonium-triazole (Sigma).

### BiFC and GO-PROMTO assay

*Agrobacterium tumefaciens* strain *AGL1* carrying the BiFC constructs and another strain generating the P19 protein to suppress gene silencing were resuspended in infiltration medium (10 mM MgCl_2_, 10 mM MES and pH 5.7) to a final concentration of OD_600_ 0.03. After 2 h of incubation at room temperature, the cells were infiltrated into the abaxial surface of *N. benthamiana* leaves using a 1-ml needleless syringe. After 62 h, leaf sectors were excised, mounted in UHQ water and imaged on an inverted Leica SP2 confocal microscope using a 63 × HCX PL Apo oil objective (numerical aperture of 1.4). Images were collected using the average of 16 linear scans and all images were taken with the same settings. Each inoculation was performed on triplicate leaves and all transformations were performed on at least two separate occasions. The nuclear marker CFP-N7 (cyan) was a generous gift from Dr Tezz Quon (Monash University, Melbourne, Australia) and was infiltrated with all combinations as a positive control to assess transformation efficiency^62^.

To confirm the membrane topology for STL1 and STL2, we used the GO-PROMTO BiFC assay^37^ and transiently expressed combinations of constructs with either cytosolic or lumenal fluorescent reporters in *N. benthamiana* leaves. The combinations that were transiently co-expressed included the STL1/2 BiFC constructs (Yn-STL1, Yn-STL2, Yc-STL1 or Yc-STL2), and the GO-PROMTO luminal (Yn-TMD and Yc-TMD) and cytosolic (TMD-Yn and TMD-Yc) reporter constructs^63^.

### Protease protection assay

For topology analysis, STL1 was expressed under its own promoter with 4 × myc tag at the C-terminus in *Arabidopsis* callus. Microsome preparation was performed from callus tissue homogenized in an equal volume of homogenization buffer (250 mM Suc, 25 mM HEPES, pH 7.5, 1 mM EDTA, 1 mM dithiothreitol and Complete Protease Inhibitor Cocktail Tablets; Roche). The homogenate was centrifuged twice for 30 min at 2,200*g*. The supernatant was centrifuged for 2 h at 4 °C at 100,000*g* onto a cushion of 18% (v/v) iodixanol in homogenization buffer. Concentrated membranes were collected from the interface^64^. For the protease assay, microsome preparations were treated with or without detergent (1% [v/v] Triton X-100) and with or without protease (1 ng μl^−1^ proteinase K (Sigma)) overnight at room temperature. Phenylmethanesulfonyl fluoride (PMSF) was added to the reactions to inactivate the protease. The proteins were denatured and reduced by the addition of 4 × Laemmli loading buffer containing DTT as reducing agent and incubation at 90 °C for 10 min. The proteins were separated by SDS-PAGE and then transferred to a polyvinylidene fluoride membrane for immunoblotting. A rabbit anti-c-Myc polyclonal IgG antibody (A-14, Santa Cruz Biotech SC-789) was used at a 1:1,000 dilution as the primary antibody, and a goat-anti-rabbit horseradish peroxidase (Bio-Rad 170–6515) was used at a 1:10,000 dilution as the secondary antibody.

### Gel electrophoresis and western blot

Total proteins were extracted for SDS-PAGE from 14-day-old GFP-CesA3-expressing seedlings or stem materials from 5-week-old plants^65^. BN-PAGE analysis for CesA complex was performed as follows^39^. One gram of samples were ground with a mortar and pestle on ice in 1 ml of extraction buffer (2 mM EGTA, 2 mM EDTA, 100 mM MOPs, pH 7.0) with the protease inhibitor cocktail (Roche). Extracts were centrifuged at 5,000 g for 10 min to remove cell debris. A crude microsomal pellet was prepared by centrifuging the supernatant at 100,000*g* for 1 h at 4 °C. The pellet was resuspended in 150 μl of the resuspension buffer (extraction buffer plus 2% (v/v) of Triton X-100) followed by incubation on ice for 30 min. The supernatant was clarified by further centrifugation at 100,000 g for 30 min at 4 °C and loaded for BN-PAGE using the NativePAGE Novex 3–12% Bis-Tris Protein Gels (Invitrogen). After electrophoresis, the gel was heated in denaturing buffer (3.3% (m/v) SDS, 65 mM Tris-HCl, pH6.8) to denature protein complexes and facilitate the transferring of protein to polyvinylidene fluoride membranes (Millipore). The membrane was washed with methanol to remove the Coomassie blue and reversibly stained with Ponceau S (Sigma) for loading control. Anti-GFP antibody (1:1,000 dilution; ThermoFisher, A-11122) was used as the primary antibody for detecting GFP-CesA3, while anti-CesA8 antibody (1:1,000 dilution; Agrisera, AS12 2580) was used for the stem samples. The band intensity was analysed with Quantity One software (Bio-Rad).

### RT-PCR and qRT-PCR

Total RNA was isolated from 10-day-old seedlings by using the RNase plant mini kit (Qiagen) and treated with DNase I (Ambion) to avoid genomic DNA contamination. First-strand cDNA was synthesized from 1.5 μg total RNA samples using the Superscript III Reverse Transcriptase (Invitrogen). The resulting cDNA was used as template for RT-PCR with a programme of 95 °C for 5 min, 35 cycles of 95 °C for 30 s, 60 °C for 30 s and 72 °C for 2.5 min, followed by 72 °C for 10 min.

The cDNA was diluted 4 times and mixed together with SYBR-Green master mix (Applied Biosystems) and primers (Supplementary Table 2) for qRT-PCR (Gene AMP 7900 Sequence Detector, Applied Biosystems, United Kingdom). The programme was 50 °C for 2 min, 95 °C for 10 min, 40 cycles of 95 °C for 15 s and 60 °C for 1 min, followed by 95 °C for 15 s and 60 °C for 15 s. The expression level was calculated by the 2^(−ΔΔCt)^ method.

### Mucilage phenotype analysis

Dried mature seeds were first imbibed in excess of millipore filtered deionized water for 1 h, then incubated in a 0.01% (w/v) ruthenium red solution for 30 min and rinsed with water. The adherent mucilage was observed with a Zeiss Axioimager M2 microscope (× 20 magnification using High quality differential interference contrast imaging). The imbibed seeds were separately stained with 0.01% (w/v) pontamine fast scarlet 4B, a cellulose-specific dye (S479896; Sigma) for 1 h followed by rinsing in water. The stained cellulose in the seed-adherent mucilage was observed with a Leica TCS SP8 confocal laser scanning microscope, the Pontamine Fast Scarlet 4B dye was excited at 561 nm and detected in the 570–640 nm range.

### Statistical analysis

A *t*-test (two tailed) was performed for statistical analysis if not otherwise specified. An F-test was used to determine equal or unequal variance in the *t*-test. *, ** and *** indicate *P* value<0.05,<0.01 and<0.001, respectively.

### Data availability

Solid-state NMR data is available at: https://www.repository.cam.ac.uk/handle/1810/254646. The authors declare that all other relevant data supporting the findings of this study are available within the article and its Supplementary Information Files or on request from the corresponding authors.

## Additional information

**How to cite this article:** Zhang, Y. *et al*. Golgi-localized STELLO proteins regulate the assembly and trafficking of cellulose synthase complexes in *Arabidopsis*. *Nat. Commun.* 7:11656 doi: 10.1038/ncomms11656 (2016).

## Supplementary Material

Supplementary Information Supplementary Figures 1-7 and Supplementary Tables 1-2

Supplementary Data 1 Annotated matlab script which uses a peak-finding algorithm to measure the object diameter of Golgi bodies in fluorescence images.

## References

[b1] Carpita N. & McCann M. in Biochemistry and Molecular Biology of Plants eds Buchanan B. B., Gruissem W., Jones R. L. 52–108American Society of Plant Physiologists (2000).

[b2] Geisler D. A., Sampathkumar A., Mutwil M. & Persson S. Laying down the bricks: logistic aspects of cell wall biosynthesis. Curr. Opin. Plant Biol. 11, 647–652 (2008). 18818118 10.1016/j.pbi.2008.08.003

[b3] McFarlane H. E., Doring A. & Persson S. The cell biology of cellulose synthesis. Annu. Rev. Plant Physiol. 65, 69–94 (2014). 10.1146/annurev-arplant-050213-040240 24579997

[b4] Taylor N. G., Howells R. M., Huttly A. K., Vickers K. & Turner S. R. Interactions among three distinct CesA proteins essential for cellulose synthesis. Proc. Natl Acad. Sci. USA 100, 1450–1455 (2003). 12538856 10.1073/pnas.0337628100 PMC298793

[b5] Desprez T. . Organization of cellulose synthase complexes involved in primary cell wall synthesis in *Arabidopsis thaliana*. Proc. Natl Acad. Sci. USA 104, 15572–15577 (2007). 17878303 10.1073/pnas.0706569104 PMC2000492

[b6] Persson S. . Genetic evidence for three unique components in primary cell-wall cellulose synthase complexes in *Arabidopsis*. Proc. Natl Acad. Sci. USA 104, 15566–15571 (2007). 17878302 10.1073/pnas.0706592104 PMC2000526

[b7] Haigler C. H. & Brown R. M. Jr Transport of rosettes from the Golgi apparatus to the plasma membrane in isolated mesophyll cells of *Zinnia elegans* during differentiation to tracheary elements in suspension culture. Protoplasma 134, 111–120 (1986).

[b8] Gardiner J. C., Taylor N. G. & Turner S. R. Control of cellulose synthase complex localization in developing xylem. Plant Cell 15, 1740–1748 (2003). 12897249 10.1105/tpc.012815 PMC167166

[b9] Atanassov I. I., Pittman J. K. & Turner S. R. Elucidating the mechanisms of assembly and subunit interaction of the cellulose synthase complex of *Arabidopsis* secondary cell walls. J. Biol. Chem. 284, 3833–3841 (2009). 19056734 10.1074/jbc.M807456200

[b10] Gutierrez R., Lindeboom J. J., Paredez A. R., Emons A. M. & Ehrhardt D. W. *Arabidopsis* cortical microtubules position cellulose synthase delivery to the plasma membrane and interact with cellulose synthase trafficking compartments. Nat. Cell Biol. 11, 797–806 (2009). 19525940 10.1038/ncb1886

[b11] Crowell E. F. . Pausing of Golgi bodies on microtubules regulates secretion of cellulose synthase complexes in *Arabidopsis*. Plant Cell 21, 1141–1154 (2009). 19376932 10.1105/tpc.108.065334 PMC2685615

[b12] Morgan J. L. W., Strumillo J. & Zimmer J. Crystallographic snapshot of cellulose synthesis and membrane translocation. Nature 493, 181–186 (2013). 23222542 10.1038/nature11744 PMC3542415

[b13] Paredez A. R., Somerville C. R. & Ehrhardt D. W. Visualization of cellulose synthase demonstrates functional association with microtubules. Science 312, 1491–1495 (2006). 16627697 10.1126/science.1126551

[b14] Bringmann M. . POM-POM2/cellulose synthase interacting1 is essential for the functional association of cellulose synthase and microtubules in *Arabidopsis*. Plant Cell 24, 163–177 (2012). 22294619 10.1105/tpc.111.093575 PMC3289571

[b15] Li S., Lei L., Somerville C. R. & Gu Y. Cellulose synthase interactive protein 1 (CSI1) links microtubules and cellulose synthase complexes. Proc. Natl Acad. Sci. USA 109, 185–190 (2012). 22190487 10.1073/pnas.1118560109 PMC3252916

[b16] Roudier F. . COBRA, an *Arabidopsis* extracellular glycosyl-phosphatidyl inositol-anchored protein, specifically controls highly anisotropic expansion through its involvement in cellulose microfibril orientation. Plant Cell 17, 1749–1763 (2005). 15849274 10.1105/tpc.105.031732 PMC1143074

[b17] Liu L. . Brittle Culm1, a COBRA-like protein, functions in cellulose assembly through binding cellulose microfibrils. PLoS Genet. 9, e1003704 (2013). 23990797 10.1371/journal.pgen.1003704 PMC3749933

[b18] Nicol F. . A plasma membrane-bound putative endo-1,4-beta-D-glucanase is required for normal wall assembly and cell elongation in *Arabidopsis*. EMBO J. 17, 5563–5576 (1998). 9755157 10.1093/emboj/17.19.5563 PMC1170885

[b19] Vain T. . The Cellulase KORRIGAN is part of the cellulose synthase complex. Plant Physiol. 165, 1521–1532 (2014). 24948829 10.1104/pp.114.241216 PMC4119035

[b20] Sanchez-Rodriguez C. . CHITINASE-LIKE1/POM-POM1 and its homolog CTL2 are glucan-interacting proteins important for cellulose biosynthesis in *Arabidopsis*. Plant Cell 24, 589–607 (2012). 22327741 10.1105/tpc.111.094672 PMC3315235

[b21] Endler A. . A mechanism for sustained cellulose synthesis during salt stress. Cell 162, 1353–1364 (2015). 26343580 10.1016/j.cell.2015.08.028

[b22] Gillmor C. S., Poindexter P., Lorieau J., Palcic M. M. & Somerville C. Alpha-glucosidase I is required for cellulose biosynthesis and morphogenesis in *Arabidopsis*. J. Cell Biol. 156, 1003–1013 (2002). 11901167 10.1083/jcb.200111093 PMC2173474

[b23] Burn J. E. . The cellulose-deficient *Arabidopsis* mutant *rsw3* is defective in a gene encoding a putative glucosidase II, an enzyme processing N-glycans during ER quality control. Plant J. 32, 949–960 (2002). 12492837 10.1046/j.1365-313x.2002.01483.x

[b24] Lukowitz W. . *Arabidopsis cyt1* mutants are deficient in a mannose-1-phosphate guanylyltransferase and point to a requirement of N-linked glycosylation for cellulose biosynthesis. Proc. Natl Acad. Sci. USA 98, 2262–2267 (2001). 11226227 10.1073/pnas.051625798 PMC30126

[b25] Kang J. S. . Salt tolerance of *Arabidopsis thaliana* requires maturation of N-glycosylated proteins in the Golgi apparatus. Proc. Natl Acad. Sci. USA 105, 7893–7893 (2008). 10.1073/pnas.0800237105 PMC2311335 18408158

[b26] Liebminger E., Grass J., Altmann F., Mach L. & Strasser R. Characterizing the link between glycosylation state and enzymatic activity of the endo-beta1,4-glucanase KORRIGAN1 from *Arabidopsis thaliana*. J. Biol. Chem. 288, 22270–22280 (2013). 23782689 10.1074/jbc.M113.475558 PMC3829318

[b27] Rips S. . Multiple N-glycans cooperate in the subcellular targeting and functioning of *Arabidopsis* KORRIGAN1. Plant Cell 26, 3792–3808 (2014). 25238750 10.1105/tpc.114.129718 PMC4213159

[b28] Usadel B. . Co-expression tools for plant biology: opportunities for hypothesis generation and caveats. Plant Cell Environ. 32, 1633–1651 (2009). 19712066 10.1111/j.1365-3040.2009.02040.x

[b29] Ruprecht C. . FamNet: a framework to identify multiplied modules driving pathway diversification in plants. Plant Physiol. 170, 1878–1894 (2016). 26754669 10.1104/pp.15.01281 PMC4775111

[b30] Turner S. R. & Somerville C. R. Collapsed xylem phenotype of *Arabidopsis* identifies mutants deficient in cellulose deposition in the secondary cell wall. Plant Cell 9, 689–701 (1997). 9165747 10.1105/tpc.9.5.689 PMC156949

[b31] Mendu V. . Subfunctionalization of cellulose synthases in seed coat epidermal cells mediates secondary radial wall synthesis and mucilage attachment. Plant Physiol. 157, 441–453 (2011). 21750228 10.1104/pp.111.179069 PMC3165890

[b32] Sullivan S. . CESA5 is required for the synthesis of cellulose with a role in structuring the adherent mucilage of *Arabidopsis* seeds. Plant Physiol. 156, 1725–1739 (2011). 21705653 10.1104/pp.111.179077 PMC3149949

[b33] Griffiths J. S. . Unidirectional movement of cellulose synthase complexes in *Arabidopsis* seed coat epidermal cells deposit cellulose involved in mucilage extrusion, adherence, and ray formation. Plant Physiol. 168, 502–520 (2015). 25926481 10.1104/pp.15.00478 PMC4453796

[b34] Voiniciuc C. . MUCILAGE-RELATED10 produces galactoglucomannan that maintains pectin and cellulose architecture in *Arabidopsis* seed mucilage. Plant Physiol. 169, 403–420 (2015). 26220953 10.1104/pp.15.00851 PMC4577422

[b35] Nishiyama Y., Langan P. & Chanzy H. Crystal structure and hydrogen-bonding system in cellulose Ibeta from synchrotron X-ray and neutron fiber diffraction. J. Am. Chem. Soc. 124, 9074–9082 (2002). 12149011 10.1021/ja0257319

[b36] Nikolovski N. . Putative glycosyltransferases and other plant Golgi apparatus proteins are revealed by LOPIT proteomics. Plant Physiol. 160, 1037–1051 (2012). 22923678 10.1104/pp.112.204263 PMC3461528

[b37] Søgaard C. . GO-PROMTO illuminates protein membrane topologies of glycan biosynthetic enzymes in the Golgi apparatus of living tissues. PLoS ONE 7, e31324 (2012). 22363620 10.1371/journal.pone.0031324 PMC3283625

[b38] Schiller M., Massalski C., Kurth T. & Steinebrunner I. The *Arabidopsis* apyrase AtAPY1 is localized in the Golgi instead of the extracellular space. BMC Plant Biol. 12, 123 (2012). 22849572 10.1186/1471-2229-12-123 PMC3511161

[b39] Wang J., Elliott J. E. & Williamson R. E. Features of the primary wall CESA complex in wild type and cellulose-deficient mutants of *Arabidopsis thaliana*. J. Exp. Bot. 59, 2627–2637 (2008). 18495638 10.1093/jxb/ern125 PMC2486462

[b40] Engel B. D. . *In situ* structural analysis of Golgi intracisternal protein arrays. Proc. Natl Acad. Sci. USA 112, 11264–11269 (2015). 26311849 10.1073/pnas.1515337112 PMC4568700

[b41] Sampathkumar A. . Patterning and lifetime of plasma membrane-localized cellulose synthase is dependent on actin organization in *Arabidopsis* interphase cells. Plant Physiol. 162, 675–688 (2013). 23606596 10.1104/pp.113.215277 PMC3668062

[b42] Luo Y. . V-ATPase activity in the TGN/EE is required for exocytosis and recycling in *Arabidopsis*. Nat. Plants 1, 15094 (2015). 27250258 10.1038/nplants.2015.94 PMC4905525

[b43] Zhu C. . The fragile Fiber1 kinesin contributes to cortical microtubule-mediated trafficking of cell wall components. Plant Physiol. 167, 780–792 (2015). 25646318 10.1104/pp.114.251462 PMC4348757

[b44] Alonso J. M. . Genome-wide insertional mutagenesis of *Arabidopsis thalian*a. Science 301, 653–657 (2003). 12893945 10.1126/science.1086391

[b45] Dettmer J., Hong-Hermesdorf A., Stierhof Y. D. & Schumacher K. Vacuolar H^+^-ATPase activity is required for endocytic and secretory trafficking in *Arabidopsis*. Plant Cell 18, 715–730 (2006). 16461582 10.1105/tpc.105.037978 PMC1383645

[b46] Latijnhouwers M. . An *Arabidopsis* GRIP domain protein locates to the trans-Golgi and binds the small GTPase ARL1. Plant J. 44, 459–470 (2005). 16236155 10.1111/j.1365-313X.2005.02542.x

[b47] Bekh-Ochir D. . A novel mitochondrial DnaJ/Hsp40 family protein BIL2 promotes plant growth and resistance against environmental stress in brassinosteroid signaling. Planta 237, 1509–1525 (2013). 23494613 10.1007/s00425-013-1859-3 PMC3664749

[b48] Uemura T. . Qa-SNAREs localized to the trans-Golgi network regulate multiple transport pathways and extracellular disease resistance in plants. Proc. Natl Acad. Sci. USA 109, 1784–1789 (2012). 22307646 10.1073/pnas.1115146109 PMC3277133

[b49] Geldner N. . Rapid, combinatorial analysis of membrane compartments in intact plants with a multicolor marker set. Plant J. 59, 169–178 (2009). 19309456 10.1111/j.1365-313X.2009.03851.x PMC4854200

[b50] Grefen C. . A ubiquitin-10 promoter-based vector set for fluorescent protein tagging facilitates temporal stability and native protein distribution in transient and stable expression studies. Plant J. 64, 355–365 (2010). 20735773 10.1111/j.1365-313X.2010.04322.x

[b51] Shimada T. L., Shimada T. & Hara-Nishimura I. A rapid and non-destructive screenable marker, FAST, for identifying transformed seeds of *Arabidopsis* thaliana. Plant J. 61, 519–528 (2010). 19891705 10.1111/j.1365-313X.2009.04060.x

[b52] Carroll A. . Complexes with mixed primary and secondary cellulose synthases are functional in *Arabidopsis* plants. Plant Physiol. 160, 726–737 (2012). 22926318 10.1104/pp.112.199208 PMC3461551

[b53] Timmers J. . Interactions between membrane-bound cellulose synthases involved in the synthesis of the secondary cell wall. FEBS Lett. 583, 978–982 (2009). 19258017 10.1016/j.febslet.2009.02.035

[b54] Wilson S. M. . Determining the subcellular location of synthesis and assembly of the cell wall polysaccharide (1,3; 1,4)-beta-d-Glucan in grasses. Plant Cell 27, 754–771 (2015). 25770111 10.1105/tpc.114.135970 PMC4558670

[b55] Tryfona T. . Structural characterization of *Arabidopsis* leaf arabinogalactan polysaccharides. Plant Physiol. 160, 653–666 (2012). 22891237 10.1104/pp.112.202309 PMC3461546

[b56] McFarlane H. E., Young R. E., Wasteneys G. O. & Samuels A. L. Cortical microtubules mark the mucilage secretion domain of the plasma membrane in *Arabidopsis* seed coat cells. Planta 227, 1363–1375 (2008). 18309515 10.1007/s00425-008-0708-2

[b57] Spitzer M., Wildenhain J., Rappsilber J. & Tyers M. BoxPlotR: a web tool for generation of box plots. Nat. Methods 11, 121–122 (2014). 24481215 10.1038/nmeth.2811 PMC3930876

[b58] Bennett A. E., Rienstra C. M., Auger M., Lakshmi K. V. & Griffin R. G. Heteronuclear decoupling in rotating solids. J. Chem. Phys. 103, 6951–6958 (1995).

[b59] Oliveira R. P. & Driemeier C. CRAFS: a model to analyze two-dimensional X-ray diffraction patterns of plant cellulose. J. Appl. Crystallogr. 46, 1196–1210 (2013).

[b60] Polikarpov I. . Set-up and experimental parameters of the protein crystallography beamline at the Brazilian National Synchrotron Laboratory. J. Synchrotron Radiat. 5, 72–76 (1998). 16687806 10.1107/S0909049597014684

[b61] Bolte S. & Cordelieres F. P. A guided tour into subcellular colocalization analysis in light microscopy. J. Microsc. 224, 213–232 (2006). 17210054 10.1111/j.1365-2818.2006.01706.x

[b62] Kaplan-Levy R. N., Quon T., O'Brien M., Sappl P. G. & Smyth D. R. Functional domains of the PETAL LOSS protein, a trihelix transcription factor that represses regional growth in *Arabidopsis thaliana*. Plant J. 79, 477–491 (2014). 24889508 10.1111/tpj.12574

[b63] Lampugnani E. R. . A glycosyltransferase from *Nicotiana alata* pollen mediates synthesis of a linear (1,5)-α-L-arabinan when expressed in *Arabidopsis*. Plant Physiol 170, 1962–1974 (2016). 26850276 10.1104/pp.15.02005 PMC4825119

[b64] Nikolovski N. . Label-free protein quantification for plant Golgi protein localization and abundance. Plant Physiol. 166, 1033–1043 (2014). 25122472 10.1104/pp.114.245589 PMC4213074

[b65] Cahoon E. B., Shanklin J. & Ohlrogge J. B. Expression of a coriander desaturase results in petroselinic acid production in transgenic tobacco. Proc. Natl Acad. Sci. USA 89, 11184–11188 (1992). 1454797 10.1073/pnas.89.23.11184 PMC50514

