# Supplementary material for: Golgi-localized STELLO proteins regulate the assembly and trafficking of cellulose synthase complexes in Arabidopsis
Source: Nat Commun. 2016 Jun 9;7:11656. doi: 10.1038/ncomms11656 (PMC4906169; doi:10.1038/ncomms11656)
Supplement: Supplementary Information — Supplementary Figures 1-7 and Supplementary Tables 1-2 [file ncomms11656-s1.pdf]

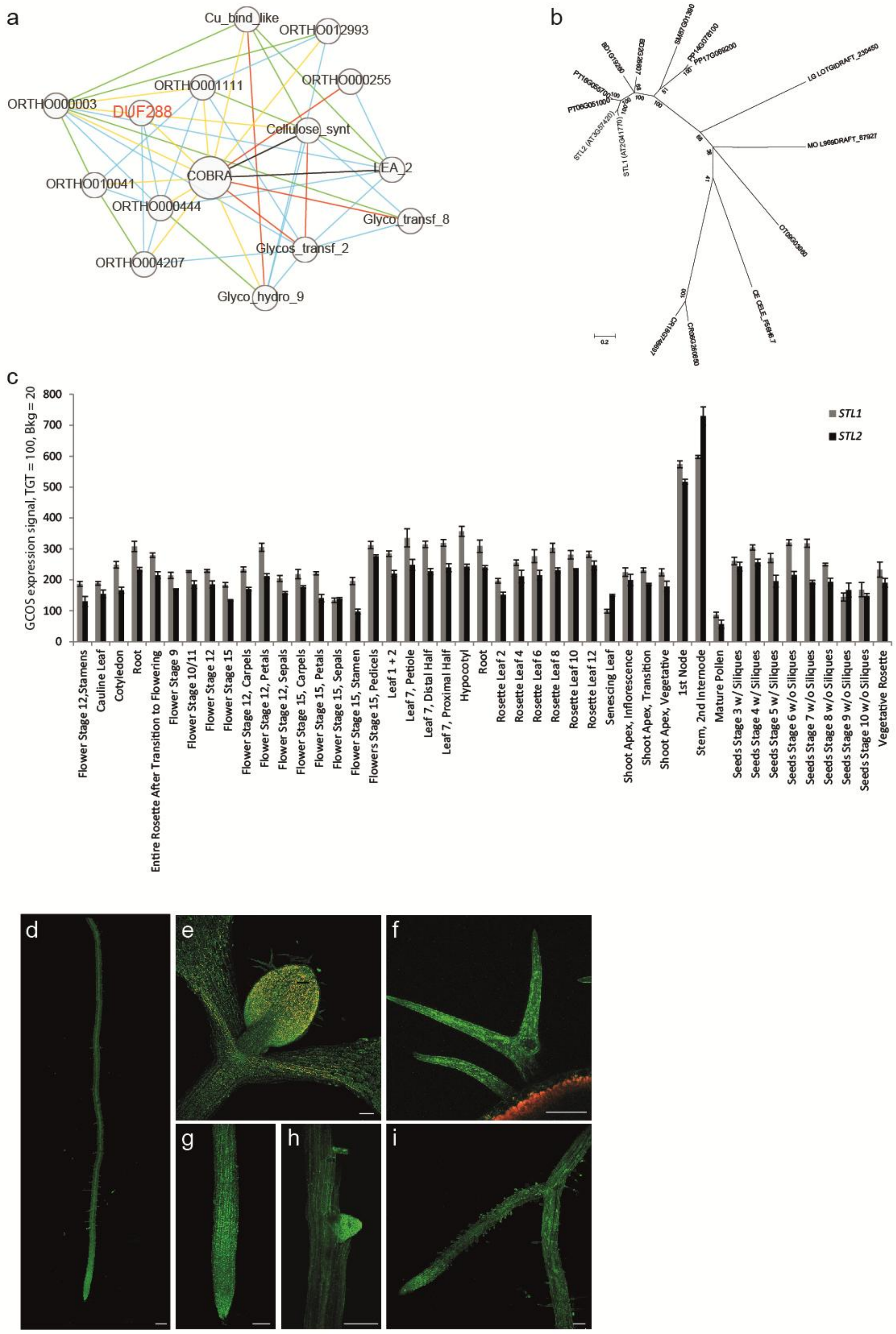

Supplementary Figure 1 Co-expression network, phylogenetic analysis and expression pattern of the STLs. (**a**) DUF288 pfam (highlighted node) is transcriptionally coordinated (edges) with several known cellulose biosynthesis related pfams. The pfam COBRA was used as search term in FamNet (http://aranet.mpimp-golm.mpg.de/famnet.html). (**b**) Phylogenetic analysis of the full length STL protein family. Amino acid sequences from *Arabidopsis*

thaliana (AT), *Populus trichocarpa* (PT), *Brachypodium distachyon* (BD), *Selaginella moellendorffii* (SM), *Physcomitrella patens* (PP), *Lottia gigantea* (LG), *Mixia osmundae* (MO), *Mortierella verticillata* (MV), *Ostreococcus tauri* (OT), *Caenorhabditis elegans* (CE) and *Chlamydomonas reinhardtii* (CR) were used for the sequence alignment and inference of the phylogenetic tree. The percentage of trees in which the associated taxa clustered together is shown next to the branches. The tree is drawn to scale, with branch lengths measured in the number of substitutions per site. (**c**) Relative expression levels of *STL1* and *STL2* across *Arabidopsis* tissues and organs (as indicated by x axis) from publicly available ATH1 Affymetrix microarray data (*Arabidopsis* eFP browser). The expression data was normalized with the GCOS (Gene Chip operating software) method with a Target intensity (TGT) and background (Bkg) threshold values of 100 and 20, respectively. Error bars represent standard deviation, most tissues were sampled in triplicate. (**d-i**) Patterns of fluorescence in *pSTL1:STL1-GFP* in *stl1* plants shows widespread expression of *STL1*.

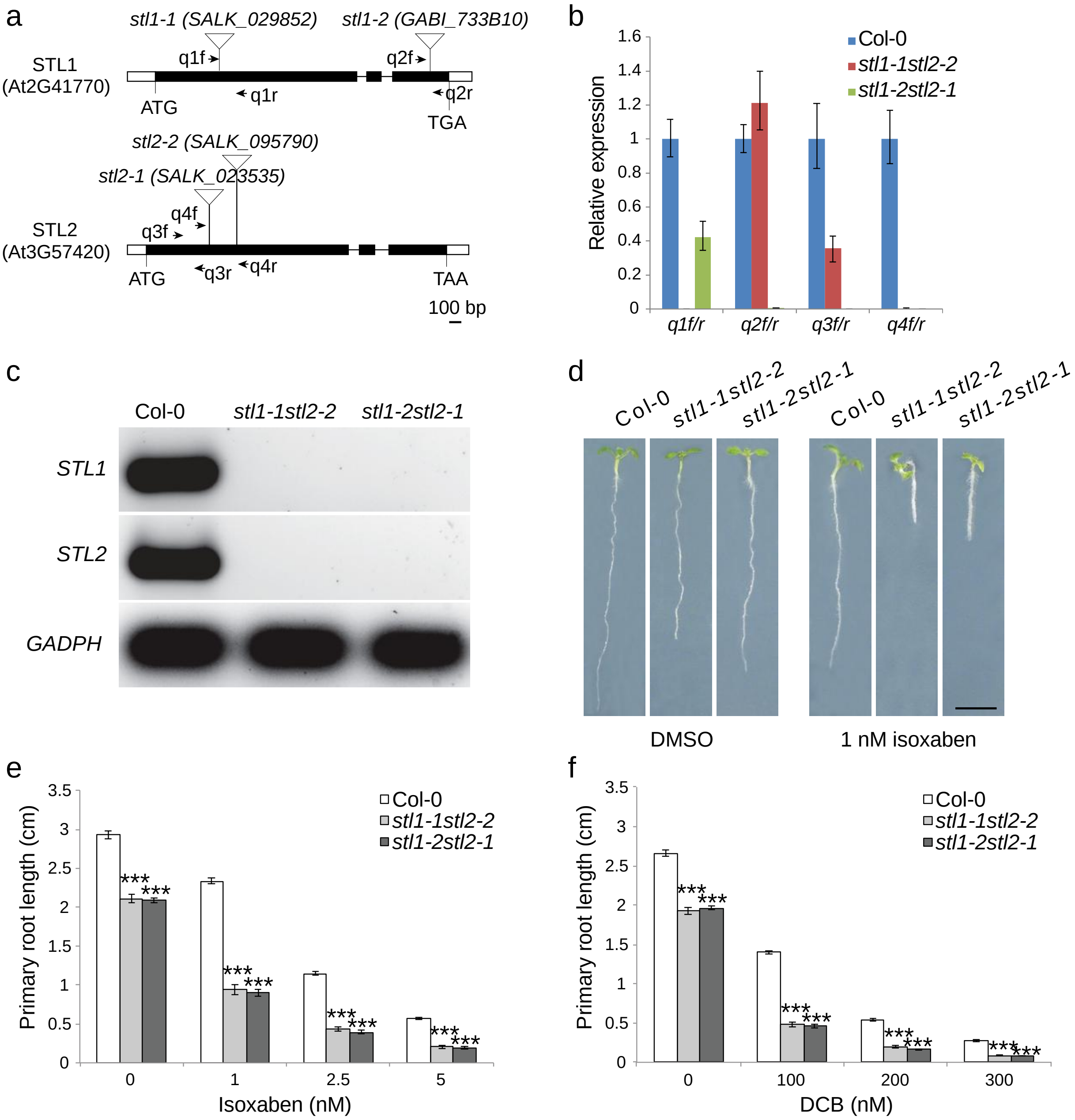

Supplementary Figure 2 Molecular and phenotypical analysis of *stl1* and *stl2* T-DNA insertional mutants. (**a**) Schematic map of the T-DNA insertion sites in the *STL1* and *STL2* gene. Black box indicates coding region; white box indicates UTR; black line indicates intron; black arrows indicates primers used in the qRT-PCR. The T-DNA was inserted 525 bp after ATG in *stl1-1* (*SALK_029852*), 177 bp before TGA in *stl1-2* (*GABI_733B10*), 510 bp after ATG in *stl2-1* (*SALK_023535*), and 750 bp after ATG in *stl2-2* (*SALK_095790*) as confirmed by PCR and sequencing. (**b**) qRT-PCR analysis of *STL1* and *STL2* transcript levels in Col-0 and *stl1stl2* mutants. Primer positions are indicated in (**a**) and Supplementary Table 2. (**c**) RT-PCR analysis for full length of *STL1* and *STL2* transcript level in Col-0 and *stl1stl2* mutants. *GADPH* was amplified and used as internal control. (**d**) Six-day-old light-grown Col-0 and *stl1stl2* mutant grown on MS media (left panel) or on MS media supplemented with 1.0 nM

isoxaben (right panel). Scale bar = 0.5 cm. (**e**) and (**f**) Bar graphs of primary root length on media supplemented with increasing concentration of isoxaben (**e**) or DCB (**f**). Values are mean (± SE) from three biological replicates with more than 10 seedlings per replicate. *** indicates p-value < 0.001, Student's t-test.

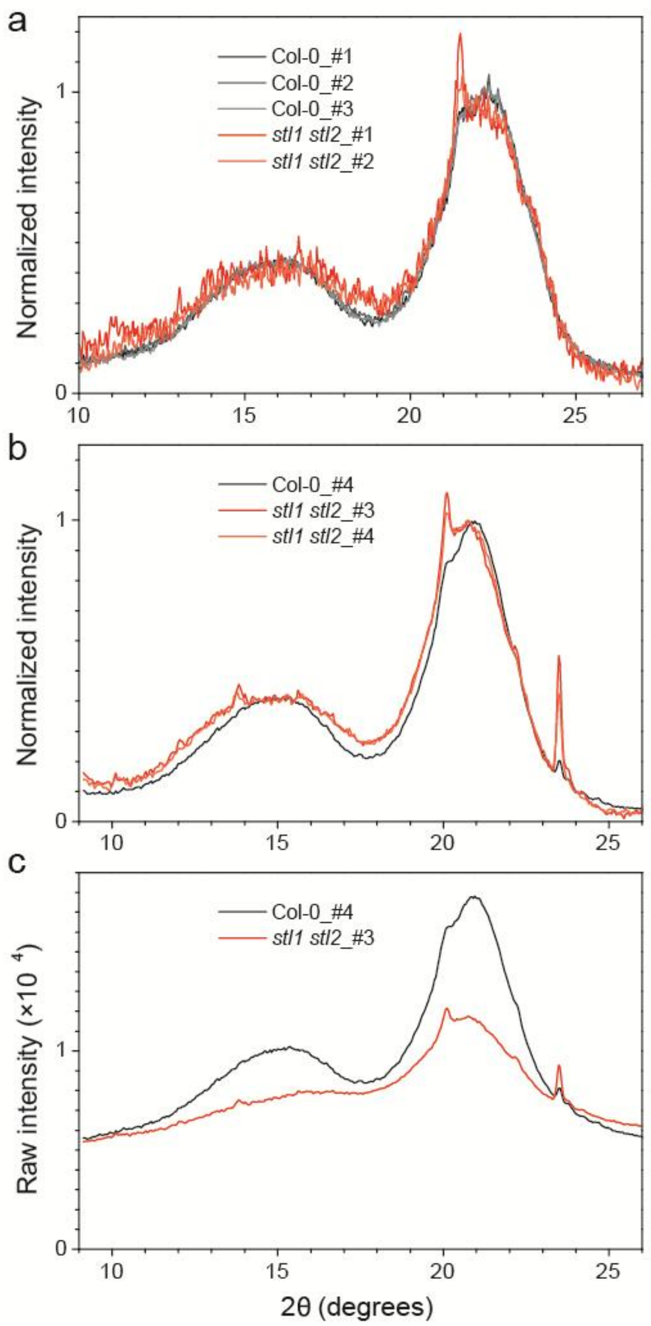

Supplementary Figure 3 X-ray fiber diffraction. Diffraction intensities along the equatorial direction. (**a**,**b**) Normalized intensity after subtraction of CRAFS-resolved polynomial background. (**a**) biological replicates used to calculate diffractograms presented in Fig. 2h. Samples were excluded from averaging if there was severe cuticular wax contamination of the cellulose XRD signal, with the height (from base to maximum) of the XRD peaks from wax far exceeding the height from the equatorial cellulose (200) peak. (**b**) Additional biological replicates characterized with higher photon flux from synchrotron beam line. (**c**) Raw intensity (before background subtraction and intensity normalization) from Col-0 and *stl1stl2* patterns shown in (**b**). The horizontal (2θ) scale in (**a**) differs from (**b**) and (**c**) because of differences in the X-ray wavelengths employed.

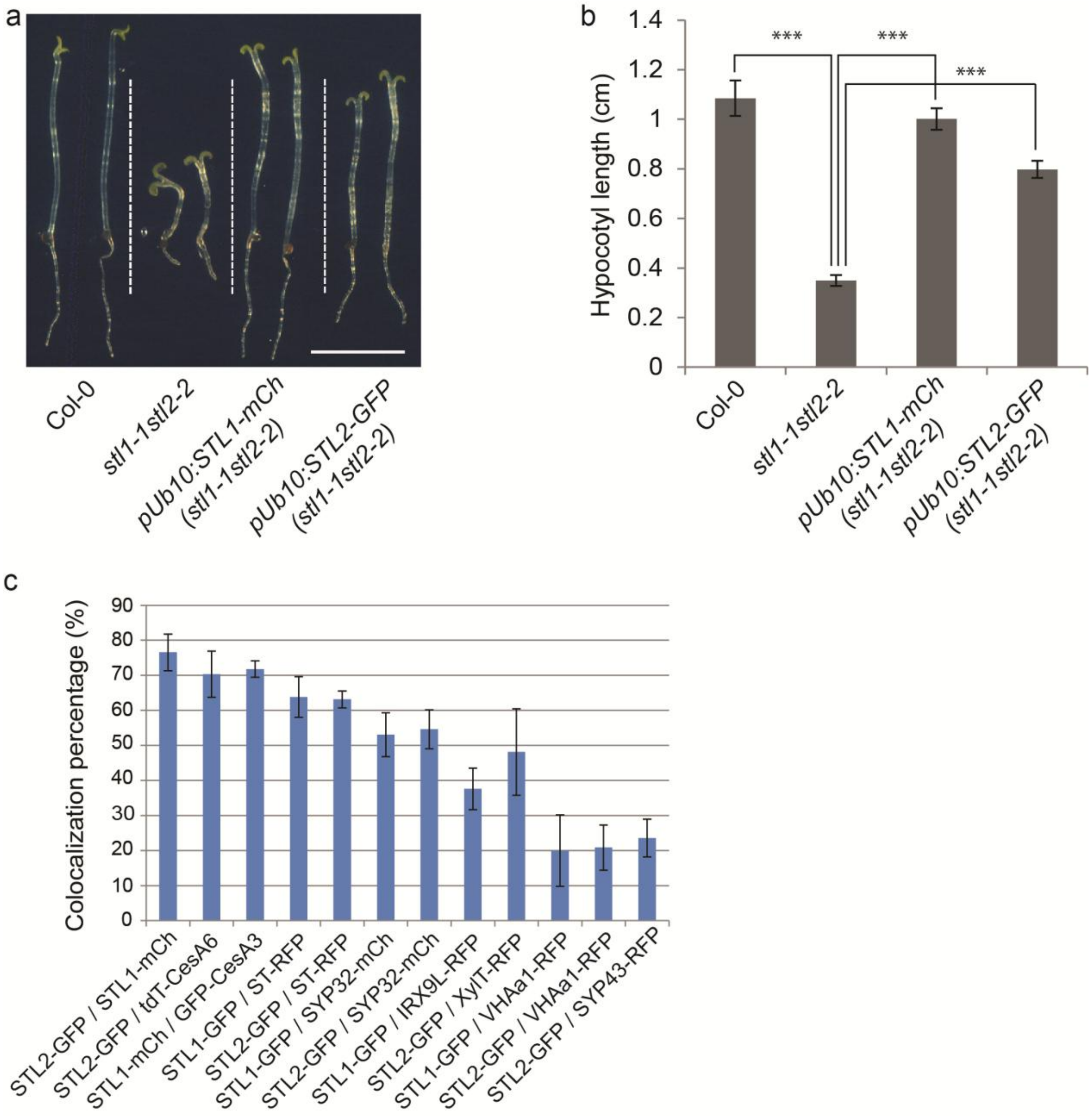

Supplementary Figure 4 Complementation of *stl1stl2* mutants with STL1-mCh and STL2-GFP constructs, and co-localization quantification data. (**a**) Six-day-old etiolated Col-0, *stl1-1stl2-2*, *pUb10:STL1-mCh(stl1-1stl2-2)* (STL1 fused C-terminally with mCherry under Ubiquitin10 promoter in the *stl1-1stl2-2* mutant background) and *pUb10:STL2-GFP(stl1-1stl2-2)* (STL2 fused C-terminally with GFP under Ubiquitin10 promoter in the *stl1-1stl2-2* mutant background) seedlings grown on medium supplemented with 0.5 nM isoxaben. Scale bar = 0.5 cm. (**b**) Bar graph for the hypocotyl length in (**a**). Values are mean (± SE) from three biological replicates with more than 10 seedlings per replicate. *** indicates p-value < 0.001, Student's t-test. (**c**) Quantitative estimates of co-localization (using Mander's coefficient) of fluorescently tagged proteins in dual-labelled seedlings as those in Figure 3. Values are the average of M1 and M2. Error bars represent SE, n ≥ 4 cells.

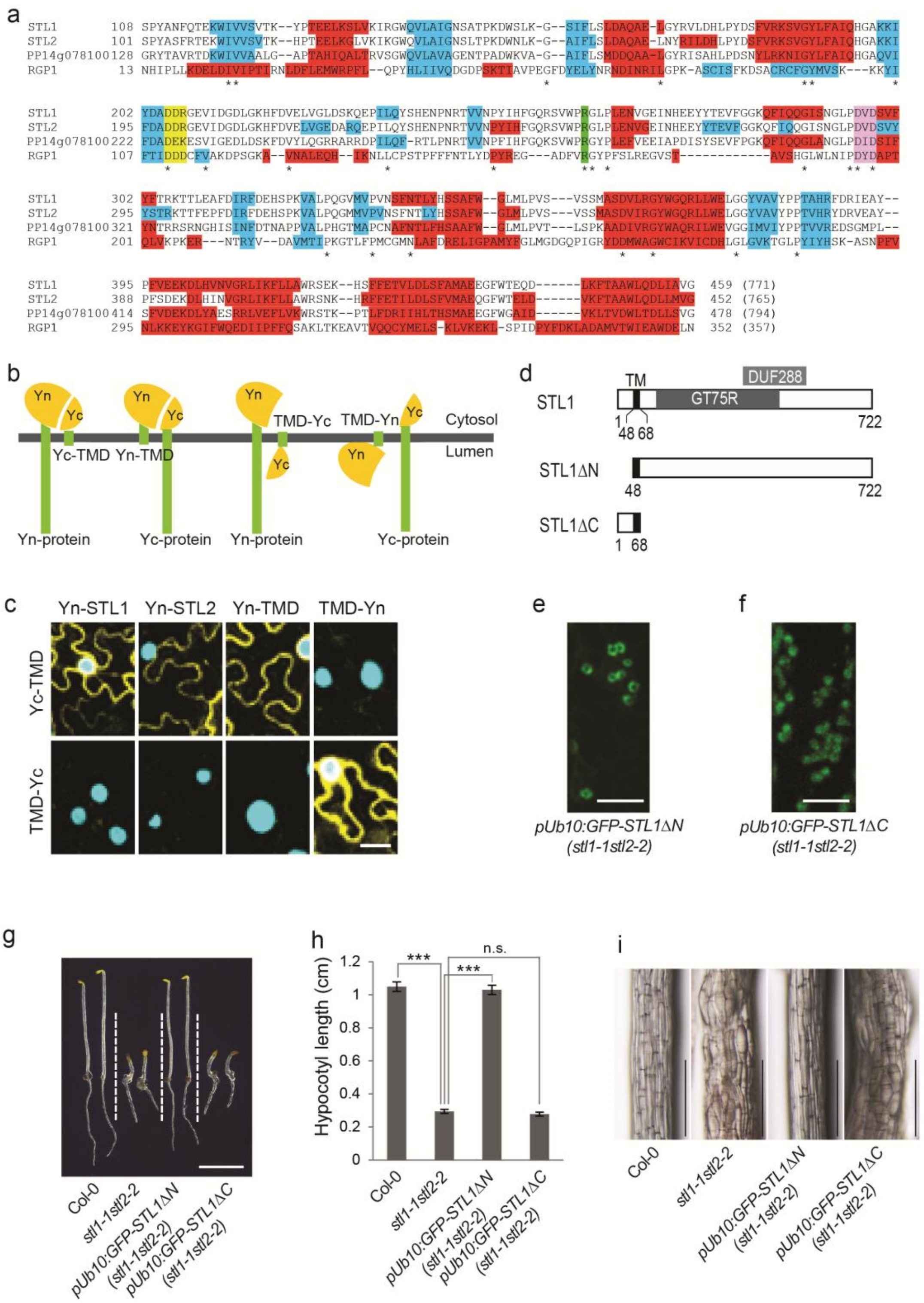

Supplementary Figure 5 Sequence alignments and topology analysis of STL1 and STL2. (**a**) Multiple sequence alignment of the GT75-related domain of *Arabidopsis* STL1, STL2, a *Physcomitrella patens* STL protein Phpat014g078100 and an *Arabidopsis* GT75 protein (RGP1). Indicated are the putative Mn^{2+}-coordinating DD motif (yellow), an additional DXD sequence (purple), and the Arg involved in self glycosylation in RGP1 (green). Predicted secondary structures, indicated in red (alpha helices) and blue (beta sheets), show some

conservation between STL and RGP proteins. (**b**) Schematic view of GO-PROMTO assays. Yn, the N-terminal part of VENUS; Yc, the C-terminal part of VENUS; TMD, the first 52 amino acids of the rat ST protein. (**c**) *N. benthamiana* epidermal leaf cells expressing Yc-TMD (cytosolic reporter) or TMD-Yc (Golgi lumen reporter) together with Yn-STLs. Yellow signal indicates that the two proteins interact and therefore are facing the same side of the Golgi membrane. The nuclear marker CFP-N7 (cyan) was included as a positive transformation control in all experiments. Scale bar = 50 μm. (**d**) Schematic diagram to show the truncated versions of STL1 protein. Numbers indicate amino acids. The STL1ΔN and STL1ΔC indicate the N-terminal truncation and C-terminal truncation, respectively, used in (**e-i**). (**e-f**) Individual images of 3-day-old etiolated hypocotyl cells expressing either GFP-STL1ΔN (**e**) or GFP-STL1ΔC (**f**). Scale bars = 5 μm. (**g**) Six-day-old etiolated Col-0, *stl1stl2*, and *stl1stl2* mutants complemented with either GFP-STL1ΔN or GFP-STL1ΔC, seedlings grown on medium supplemented with 0.5 nM isoxaben. Note that the GFP-STL1ΔN construct fully complemented the *stl1stl2* mutant phenotype. (**h**) Hypocotyl length for seedlings as those shown in (**g**). Values are mean (± SE) from three biological replicates with more than 10 seedlings per replicate. *** indicates p-value < 0.001 from Student's t-test; n.s., not significant by Student's t-test. (**i**) Close-up of hypocotyl cells of seedlings grown as in (**g**) using stereo microscopy. Scale bars = 200 μm.

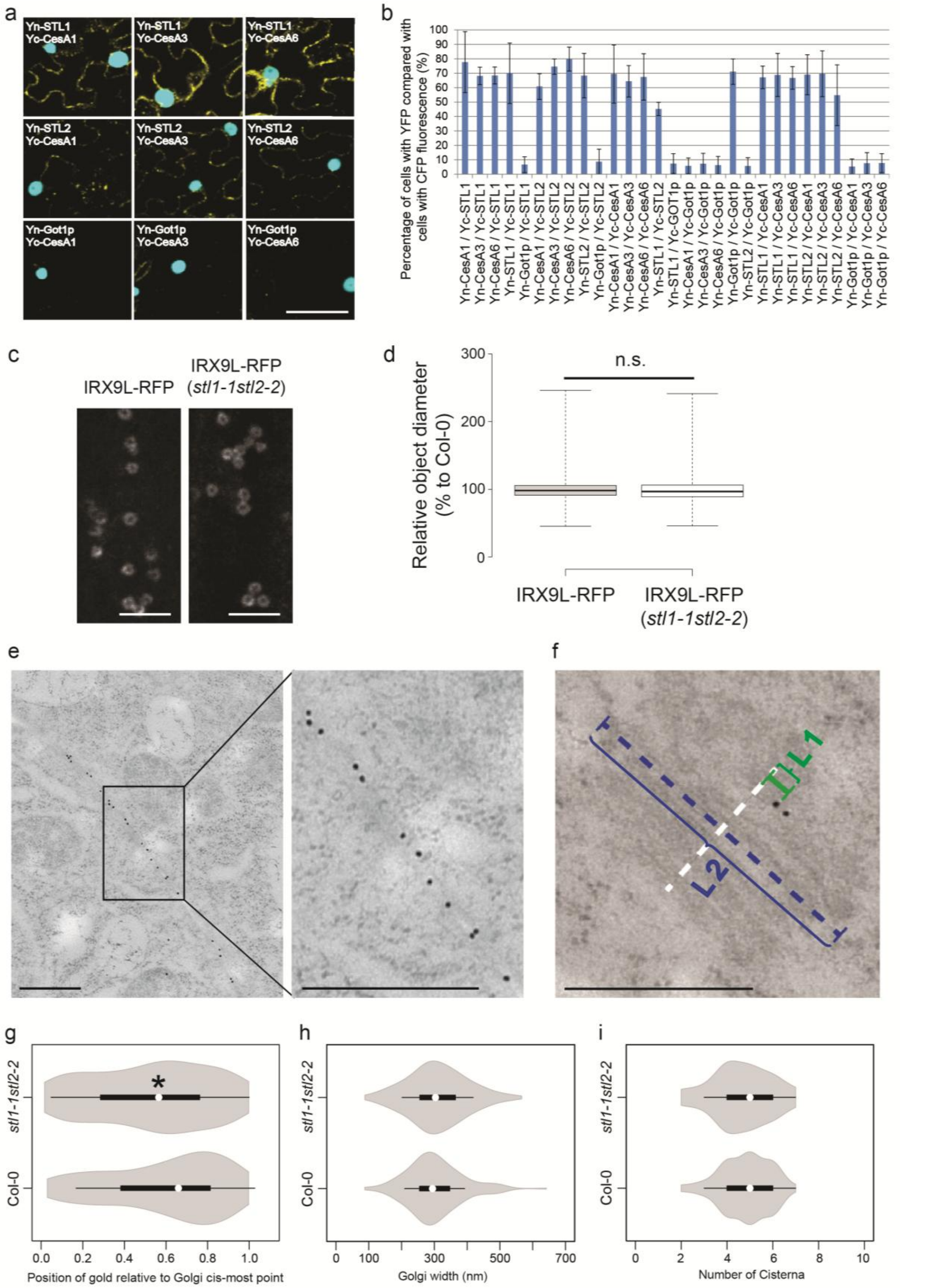

Supplementary Figure 6 BiFC assay, relative object diameter of IRX9L-RFP and electron microscopy of the Golgi. (**a**) BiFC assays detecting the interaction of CesAs and STLs in *N.benthamiana* epidermal leaf cells. The N-terminal (Yn) or C-terminal (Yc) part of VENUS was fused in frame with CesA1, CesA3, CesA6, STL1, STL2 and Got1p (negative

control), respectively. The combination of constructs is indicated in each figure panel. The nuclear marker CFP-N7 (cyan) was included as a positive transformation control in all experiments. Scale bar = 50 μm. (**b**) Quantification of percentage of cells with YFP signal compared with cells with CFP fluorescence. Values are mean (± SD), n ≥ 4 images per combination. (**c**) Fluorescent images of IRX9L-RFP. Scale bars = 5 μm. (**d**) Relative object diameter of IRX9L-RFP. Center lines show the medians; box limits indicate the 25th and 75th percentiles; whiskers extend to minimum and maximum values. For control, n = 203 Golgi from 8 cells (4 plants); for *stl1-1slt2-2*, n = 170 Golgi from 6 cells (3 plants). n.s., not significant by Student's t-test. (**e**) Representative immuno-TEM image for GFP-CesA3 signal at plasma membrane in wild-type plants. The high concentration of GFP-CesA3 in the plasma membrane demonstrates the specificity of the signal. Scale bars = 500 nm. (**f**) Schematic measurement of position of gold relative to Golgi center in Figure 7g. Position of gold relative to Golgi center was calculated as L1 divided by L2. (**g**) Violin plots for Golgi cis-trans gold distribution. Values show the distance of each gold particle from the cis-most point of the Golgi, relative to Golgi width. n ≥ 190 gold particles per genotype from two independent immuno-TEM experiments. * indicates p-value < 0.05, Student's t-test. (**h**) Violin plots of Golgi width measurements. Note there is no significant difference between the wild-type and *stl1stl2* plants (p = 0.34, Student's t-test). n = 200 Golgi per genotype from three independent cryofixation experiments. (**i**) Violin plots of Golgi Cisternae number quantification. Note there is no significant difference between the wild-type and *stl1stl2* plants (p = 0.30, Student's t-test). n = 98 Golgi per genotype from three independent cryofixation experiments. In (**g-i**), white circles show the medians; box limits indicate the 25th and 75th percentiles; whiskers extend 1.5 times the interquartile range from the 25th and 75th percentiles; polygons represent density estimates of data and extend to extreme values.

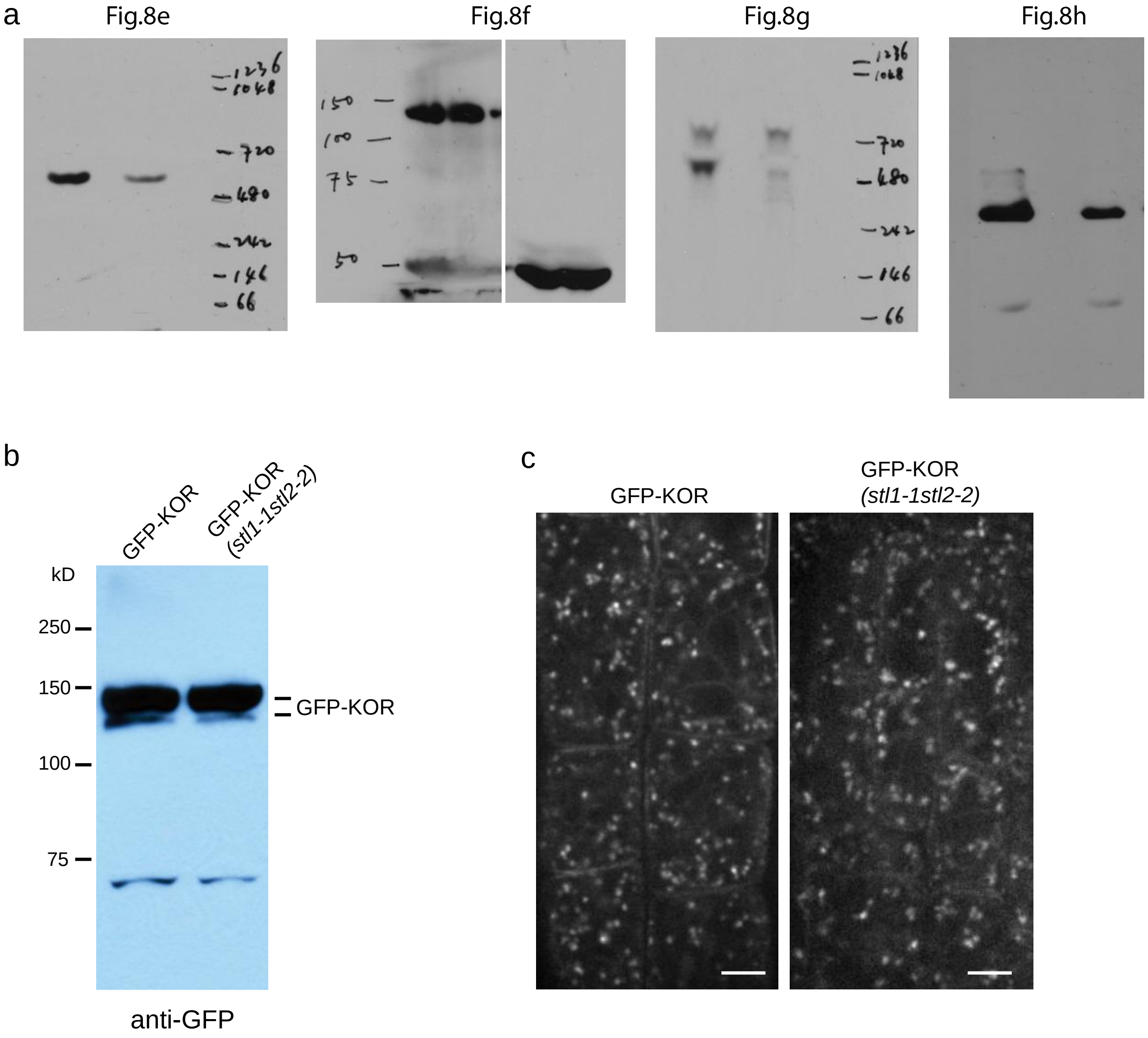

Supplementary Figure 7 Whole gel images of western blot results and glycosylation analyses of KORRIGAN **in the *stl1stl2* mutants.** (**a**) Whole gel images of western blot results in Figures 8e-h. (**b**) SDS-PAGE and western blot of GFP- KORRIGAN (KOR). Note there is no size difference of GFP-KOR in wild-type and *stl1stl2* mutants. (**c**) Sub-cellular localization of GFP-KOR in wild-type and *stl1stl2* mutants.

Supplementary Table 1. Neutral sugar and uronic acids composition of TFA treated alcohol-insoluble residues.

Tissue	Plant line	Neutral sugars (mol%)							Uronic acids (mol%)	
		Fuc	Rha	Ara	Gal	Glc	Xyl	Man	GalU	GluU
Etiolated seedlings	Col-0	1.01 ± 0.05	8.71 ± 0.30	12.32 ± 0.21	24.79 ± 1.21	8.08 ± 0.58	12.21 ± 0.17	4.42 ± 0.78	25.17 ± 1.39	3.30 ± 0.03
	stl1-1	0.97 ± 0.19	9.14 ± 0.48	12.79 ± 0.37	23.91 ± 1.41	8.70 ± 0.71	11.69 ± 0.43	3.81 ± 1.37	26.19 ± 1.29	2.79 ± 0.87
	stl2-2	0.95 ± 0.05	9.30 ± 0.36	11.95 ± 0.58	24.67 ± 1.40	8.61 ± 0.14	11.51 ± 0.32	4.51 ± 0.25	25.26 ± 0.69	3.24 ± 0.10
	stl1-1stl2-2	0.87 ± 0.11	8.41 ± 0.27	14.20 ± 0.71	26.31 ± 0.64	6.71 ± 0.02	**9.45 ± 0.36**	3.47 ± 0.45	27.93 ± 1.75	2.65 ± 0.56
	stl1-2stl2-1	0.76 ± 0.10	8.61 ± 0.21	13.61 ± 0.42	26.87 ± 1.06	7.01 ± 0.49	**9.70 ± 0.08**	4.00 ± 0.16	26.97 ± 1.62	2.46 ± 0.60
	pSTL1:STL1-GFP(stl1-1stl2-2)	0.91 ± 0.05	8.67 ± 0.18	12.80 ± 0.12	24.70 ± 1.07	9.07 ± 0.81	11.60 ± 0.27	4.67 ± 0.43	24.90 ± 0.71	**2.67 ± 0.07**
Stems	Col-0	0.74 ± 0.11	4.82 ± 0.65	3.96 ± 0.33	7.44 ± 2.83	15.29 ± 5.50	40.63 ± 12.25	3.11 ± 0.85	21.24 ± 5.54	2.77 ± 0.96
	stl1-1stl2-2	0.66 ± 0.18	4.39 ± 0.32	4.66 ± 0.90	6.99 ± 2.57	14.33 ± 3.96	40.95 ± 8.54	2.79 ± 1.00	22.72 ± 4.40	2.50 ± 1.14
Callus	Col-0	1.03 ± 0.16	4.94 ± 0.98	18.60 ± 1.77	11.54 ± 0.60	45.38 ± 1.67	8.26 ± 2.03	2.44 ± 0.29	8.67 ± 2.00	0.28 ± 0.08
	stl1-2stl2-1	1.16 ± 0.42	5.70 ± 2.00	18.25 ± 6.59	11.77 ± 3.44	42.20 ± 16.79	8.33 ± 1.10	2.52 ± 0.93	9.68 ± 2.33	0.38 ± 0.13

Values are shown ± SD n=4 (etiolated seedlings); n=3 (stems, callus)
Bold indicates significant difference p value <0.01,
Student's t test.

Supplementary Table 2. Primers used in this paper.

Primer Name	Nucleotide Sequence	Use
STL1_for	caccATGTTGGTTCAGGATCGTGC	Cloning of STL1 and STL1∆C into gateway vectors, RT-PCR
STL1_rev	TACCAACTCCATCAGAAGCGGGTC	Cloning of STL1 into gateway vectors, RT-PCR
STL2_for	caccATGTTGGTTCAAGATCGTGTGGCTC	Cloning of STL2 into gateway vectors, RT-PCR
STL2_rev	TACCAATTCCATCAATAGCGGATCAC	Cloning of STL2 into gateway vectors, RT-PCR
STL1_∆N_for	caccCTCTCTAGAATTGCTGTCTTCTCTCTCTT	Cloning of STL1∆N into gateway vectors
STL1_∆N_rev	TCATACCAACTCCATCAGAAGCG	Cloning of STL1∆N into gateway vectors
STL1_∆C_rev	tcaGTAGAGGAAGAAGAAAGCTACTATGGTGA	Cloning of STL1∆C into gateway vectors
pSTL1_for	ACGAATGGGCCCCAAAGAAAAACAAAACCCAC	Cloning of pSTL1::STL1-mGFP
pSTL1_rev	tatctgCTGCAGTACCAACTCCATCAGAAGCGGGTCGCC	Cloning of pSTL1::STL1-mGFP
mGFP_for	atcgatctgcagAGTAAAGGAGAAGAACTTTTCACTGGAGTTGTCCCA	Cloning of pSTL1::STL1-mGFP
mGFP_rev	gggaaGCGGCCGCTTATTTGTATAGTTCATCCATGCCATGTGTAATCC	Cloning of pSTL1::STL1-mGFP
NOS_for	gaataggcggccgcTTCCCCGATCGTTCAAACATTTGGCAATAAAGTT	Cloning of pSTL1::STL1-mGFP
NOS_rev	tatctggagctcGATCTAGTAACATAGATGACACCGCGCGCGA	Cloning of pSTL1::STL1-mGFP
STL1_pTFB1_for	CATGGAGGCCTTATGTTGGTTCAGGATCGTGCGG	Cloning of vectors for split-ubiquitin assays
STL1_pTFB_rev	CATGACTAGTGATCATACCAACTCCATCAGAAGCGG	Cloning of vectors for split-ubiquitin assays
STL2_pTFB1_for	CATGGAGGCCTTATGTTGGTTCAAGATCGTGTGGCTC	Cloning of vectors for split-ubiquitin assays
STL2_pTFB1_rev	CATGACTAGTGATTATACCAATTCCATCAATAGCGGATCAC	Cloning of vectors for split-ubiquitin assays
Got1p_pADSL_Nx_for	agattacgct<u>ggatcc</u>ATGGCTTCCTTGGAGATGAATGACC	Cloning of vectors for split-ubiquitin assays
Got1p_pADSL_Nx_rev	gcttgatatc<u>gaattc</u>tcTTAGACAGGGACACGCCTTCCAC	Cloning of vectors for split-ubiquitin assays
CesA1_BiFC_for	atctggaggaggaGGCgccATGGAGGCCAGTGCCGGCTT	Cloning of vectors for BiFC and GO-PROMTO assays
CesA1_BiFC_rev	AAGCAGGACTCTAGAGgatccCTAAAAGACACCTCCTTTGCCAT	Cloning of vectors for BiFC and GO-PROMTO assays
CesA3_BiFC_for	atctggaggaggaGGCgccATGGAATCCGAAGGAGAAACC	Cloning of vectors for BiFC and GO-PROMTO assays
CesA3_BiFC_rev	AAGCAGGACTCTAGAGgatccTCAACAGTTGATTCCACATTCCA	Cloning of vectors for BiFC and GO-PROMTO assays

CesA6_BiFC_for	atctggaggaggaGGCgccATGAACACCGGTGGTCGGTT	Cloning of vectors for BiFC and GO-PROMTO assays
CesA6_BiFC_rev	AAGCAGGACTCTAGAGgatccTCACAAGCAGTCTAAACCACAGATC	Cloning of vectors for BiFC and GO-PROMTO assays
STL1_BiFC_for	TCTGGAGGAGGAGGCGCCATGTTGGTTCAG	Cloning of vectors for BiFC and GO-PROMTO assays
STL1_BiFC_rev	AGCAGGACTCTAGAGTCATACCAACTC	Cloning of vectors for BiFC and GO-PROMTO assays
STL2_BiFC_for	TCTGGAGGAGGAGGCGCCATGTTGGTTC	Cloning of vectors for BiFC and GO-PROMTO assays
STL2_BiFC_rev	AGCAGGACTCTAGAGTTATACCAATTCC	Cloning of vectors for BiFC and GO-PROMTO assays
Got1p_BiFC_for	TCTGGAGGAGGAGGCGCCATGGCTTCCTTGGAGATG	Cloning of vectors for BiFC and GO-PROMTO assays
Got1p_BiFC_rev	AGCAGGACTCTAGAGTTAGACAGGGACACGCC	Cloning of vectors for BiFC and GO-PROMTO assays
q1f	GCTGAATTGGGTTATCGAGTTTTAGATCAC	qRT-PCR
q1r	CGGAACGTTGCCCGAAATGA	qRT-PCR
q2f	GGTATTCTATGTGCCTAAAAGACTGGTAACTGA	qRT-PCR
q2r	CAGGTGCTTTAGCAGAATACAGACTCGA	qRT-PCR
q3f	AAACCTCACCTTACGCTAGTTTCCGTACA	qRT-PCR
q3r	ATGATCCAAAATCCGGTAATTAAGCTCAG	qRT-PCR
q4f	CTGAGCTTAATTACCGGATTTTGGATCAT	qRT-PCR
q4r	TCTCTAATGGCAAACCTCTAGGCCAA	qRT-PCR
GADPH_for	TTGGTGACAACAGGTCAAGCA	qRT-PCR, RT-PCR
GADPH_rev	AAACTTGTCGCTCAATGCAATC	qRT-PCR, RT-PCR
stl1-1_LP	TTCACTTGCAAACAAAACACG	genotyping the T-DNA insertion lines
stl1-1_RP	ACAGCAACATAACCACCAAGC	genotyping the T-DNA insertion lines
stl1-2_LP	AATTTTGGGAATGTTGTGCTG	genotyping the T-DNA insertion lines
stl1-2_RP	GGATCTCTTCACCAAGCTTCC	genotyping the T-DNA insertion lines
stl2-1_LP	ATAAGCAACCTCCAAATTCCG	genotyping the T-DNA insertion lines
stl2-1_RP	ACGCAGAGGAATGGTACAATG	genotyping the T-DNA insertion lines
stl2-2_LP	CAAGATCGTGTGGCTCCTAAG	genotyping the T-DNA insertion lines
stl2-2_RP	CTCCAAGCAAGCAAGAATTTG	genotyping the T-DNA insertion lines
